# Cell-cell communication through FGF4 generates and maintains robust proportions of differentiated cell types in embryonic stem cells

**DOI:** 10.1242/dev.199926

**Published:** 2021-11-05

**Authors:** Dhruv Raina, Azra Bahadori, Angel Stanoev, Michelle Protzek, Aneta Koseska, Christian Schröter

**Affiliations:** Department of Systemic Cell Biology, Max Planck Institute of Molecular Physiology, 44227 Dortmund, Germany

**Keywords:** FGF signaling, Embryonic stem cells, Preimplantation development, Primitive endoderm, Epiblast, Dynamical system, Mouse

## Abstract

During embryonic development and tissue homeostasis, reproducible proportions of differentiated cell types are specified from populations of multipotent precursor cells. Molecular mechanisms that enable both robust cell-type proportioning despite variable initial conditions in the precursor cells, and the re-establishment of these proportions upon perturbations in a developing tissue remain to be characterized. Here, we report that the differentiation of robust proportions of epiblast-like and primitive endoderm-like cells in mouse embryonic stem cell cultures emerges at the population level through cell-cell communication via a short-range fibroblast growth factor 4 (FGF4) signal. We characterize the molecular and dynamical properties of the communication mechanism and show how it controls both robust cell-type proportioning from a wide range of experimentally controlled initial conditions, as well as the autonomous re-establishment of these proportions following the isolation of one cell type. The generation and maintenance of reproducible proportions of discrete cell types is a new function for FGF signaling that might operate in a range of developing tissues.

## Abstract (precis)

**Summary:** Experiments in embryonic stem cells reveal how the coupling of cell fate decisions in a population leads to the self-organized differentiation of stable ratios of cell types.

## INTRODUCTION

The differentiation of specialized cell types from populations of multipotent precursor cells is the basis of embryonic development and tissue homeostasis in the adult. Developing tissues generally produce and maintain a standard end result consisting of defined proportions of differentiated cell types despite biological noise and perturbations, a behavior termed ‘canalization’ (Waddington, 1942). Molecular mechanisms that contribute to canalized development by controlling the proportions of differentiated cell types remain to be characterized.

Mammalian preimplantation development is a prime example of developmental canalization. The size of the three lineages involved [trophectoderm (TE), epiblast (Epi) and primitive endoderm (PrE)] is remarkably constant between mouse preimplantation embryos (Saiz et al., 2016). Furthermore, mammalian embryos can regulate the proportions of these three lineages following splitting, fusing or the addition of embryonic stem cells (ESCs), such that the embryo is capable of post-implantation development (Arias et al., 2013; Bedzhov et al., 2014; Bradley et al., 1984; Gardner, 1968; Tarkowski, 1959, 1961). The differentiation of Epi and PrE cells from inner cell mass (ICM) cells is controlled by transcription factors, such as NANOG and GATA6, which mark and specify Epi and PrE cells, respectively. These factors are initially co-expressed in ICM cells and become mutually exclusive as cells differentiate (Chazaud et al., 2006; Plusa et al., 2008; Simon et al., 2018). In addition, PrE differentiation requires fibroblast growth factor (FGF)/extracellular regulated kinase (ERK) signaling (Chazaud et al., 2006; Kang et al., 2017; Krawchuk et al., 2013; Molotkov et al., 2017). Current models for cell differentiation in the ICM posit that mutually repressive interactions between transcription factors together with heterogeneous FGF/ERK signaling allocate individual cells to one of the two lineages (Bessonnard et al., 2014; De Caluwé et al., 2019; Chickarmane and Peterson, 2008; De Mot et al., 2016; Yamanaka et al., 2010). More recently, FGF signaling has been shown to regulate Epi and PrE lineage sizes following the addition or ablation of cells, suggesting that it orchestrates cell differentiation at the population level (Saiz et al., 2020).

Population-level mechanisms for cell differentiation are an attractive solution to the problem of developmental canalization, because it has been shown theoretically that populations of communicating cells can re-establish specific cell-type proportions following perturbations (Stanoev et al., 2021). This theory furthermore predicts that the differentiation outcomes are insensitive to initial conditions, because the heterogeneous cell identities represent a collective state that is generated and maintained at the level of the communicating population, rather than being specified intrinsically in each cell. However, identifying a molecular mechanism that leads to such emergent phenomena requires an experimental system in which both the initial conditions in the precursor population as well as cell-cell communication can be precisely controlled.

**Fig. 1.**
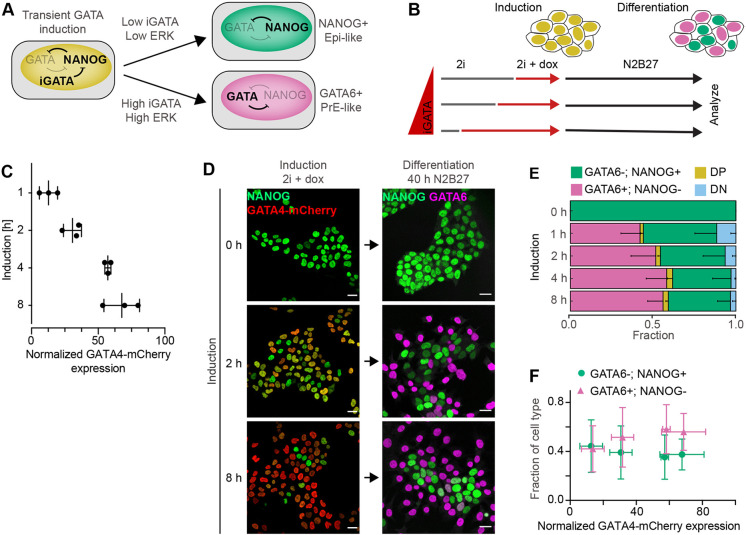
**Proportions of differentiated cell types are independent from GATA4-mCherry induction levels.** (A) Schematic of cell differentiation following transient doxycycline-controlled expression of inducible GATA factors (iGATA). (B) Experimental protocol for titrating inducible GATA4-mCherry expression levels by doxycycline addition to individual samples at different time points. The total time from seeding to analysis is held constant. (C) GATA4-mCherry expression levels for different durations of doxycycline induction in 2i+LIF medium measured by flow cytometry, normalized to the non-induced control. Individual data points show mean fluorescence intensities from at least 20,000 cells in an individual experiment, bars indicate mean±s.d. across *n*=3 independent experiments. (D) Left: immunostaining for NANOG (green) and GATA4-mCherry (red) in inducible cell lines immediately after the end of a doxycycline pulse of the indicated durations. Right: immunostaining for NANOG (green) and GATA6 (magenta) in cells treated with doxycycline for the indicated durations, followed by 40 h of differentiation in N2B27 medium. Cells without doxycycline induction had been continuously maintained in 2i medium. (E) Average cell-type proportions from *n*=4 independent experiments; the fraction of GATA6+; NANOG− cells is in magenta, GATA6−; NANOG+ cells is in green, double-positive cells (DP) is in yellow and double-negative cells (DN) is in blue. Error bars show 95% confidence intervals (CI). (F) Plot of average proportions of GATA6+; NANOG− cells (magenta) and GATA6−; NANOG+ cells (green) versus mean GATA4-mCherry levels for different doxycycline induction times. Individual datapoints correspond to different induction times, horizontal bars indicate ±s.d., vertical bars indicate 95% confidence interval. Scale bars: 20 µm.

The specification of Epi- and PrE-like cells from ESCs following the transient expression of exogenous GATA factors is a suitable model system to investigate mechanisms of cell-type proportioning. In serum-containing medium, the specification of PrE-like cells requires above-threshold levels of inducible GATA factors and ERK activity, consistent with a model in which mutually repressive interactions between NANOG and GATA factors together with FGF/ERK signaling control differentiation at the single cell level (Fig. 1A) (Schröter et al., 2015). To explore the role of population-level mechanisms, we studied cell differentiation in defined serum-free media in which signaling cues are produced solely by the cells themselves. Under these conditions, robust proportions of Epi- and PrE-like cells differentiate from a wide range of initial conditions generated by experimentally controlled GATA expression levels, and regenerate from populations of purified PrE-like cells. We use communication-deficient mutant cell lines and simulations to demonstrate that cell-cell communication via a short-range FGF4 signal is the minimal molecular mechanism underlying this robust population-level behavior. These results provide evidence that local cell-cell communication via a secreted growth factor can contribute to the differentiation of reproducible global proportions of specialized cell types.

## RESULTS

### Differentiation of robust proportions of Epi- and PrE-like cells in ESC cultures

To study population-level mechanisms that control the proportions of PrE-like and Epi-like cells in ESC populations, we used ESC lines carrying doxycycline-inducible GATA4-mCherry constructs that were integrated via PiggyBac transgenesis and that allowed sampling of a wide range of GATA4-mCherry expression levels. Cells were kept in chemically defined minimal N2B27 medium supplemented with the MEK inhibitor PD0325901 (PD03), the GSK3 inhibitor CHIR99021 and the cytokine LIF (2i+LIF medium; Ying et al., 2008) during routine culture and transgene induction to maintain pluripotency. To initiate differentiation, cells were switched to N2B27 only by simultaneously removing doxycycline, LIF and inhibitors (Fig. 1B).

Under these conditions, an 8 h pulse of GATA4-mCherry expression triggered the rapid downregulation of NANOG and the upregulation of endogenous GATA6 (Fig. S1A,B). Although some cells co-expressed NANOG and GATA6 8 h after the end of the GATA4-mCherry pulse, mutually exclusive expression of GATA6 and NANOG was established within 16 h and became more pronounced until 40 h (Fig. S1A-C). From this point onwards, the proportion of GATA6-positive cells decreased and the proportion of GATA6-negative increased (Fig. S1D). The separation of the two cell populations in the NANOG;GATA6 expression space suggests that this shift in proportions beyond 40 h is a consequence of differential proliferation rates rather than of fate transitions in individual cells. NANOG expression levels in the GATA6-negative cell cluster decreased over time, consistent with differentiation along the embryonic epiblast lineage (Fig. S1C). At 40 h, expression of the three PrE-markers GATA6, SOX17 and laminin was mutually exclusive with NANOG expression (Niakan et al., 2010, Fig. S1E,F). Thus, transient expression of GATA4-mCherry followed by 40 h of differentiation in defined, growth factor-free medium subdivided an initially homogeneous culture into two cell types with Epi- and PrE-like characteristics.

To determine how the proportions of the two cell types were affected by expression levels of the inducible GATA4-mCherry protein, we titrated GATA4-mCherry expression by staggering the initiation of doxycycline induction in time, while keeping the duration of the entire experiment constant (Fig. 1B). GATA4-mCherry levels increased, and NANOG expression levels decreased with longer doxycycline induction time, both in the population and in individual cells (Fig. 1C,D; Fig. S2A). However, despite these different transcription factor expression levels at the start of differentiation, we observed similar proportions of both GATA6+;NANOG− PrE-like and GATA6-;NANOG+ Epi-like cells 40 h later (Fig. 1D; Fig. S2B). The proportion of PrE-like cells slightly increased, from 43.6±12.1% for 1 h to 59.7±11.9% (*P*=0.02) and 57.2±8.7% (*P*=0.04) for 4 h and 8 h of induction, respectively, but did not show any differences between all other conditions (*P*>0.05, Tukey's multiple comparisons test). The proportion of Epi-like cells was stable for different induction times (*P*>0.05) (Fig. 1E; Fig. S2B). Thus, in minimal medium, a wide range of inducible GATA4-mCherry expression levels leads to similar proportions of differentiated cell types (Fig. 1F). This robust proportioning of cell types could be a population-level phenomenon or, alternatively, could have a cell-intrinsic basis, such as the prespecification of cell types or a limited differentiation potential in a subset of cells. To rule out this latter possibility, we promoted ERK activity by adding recombinant FGF4 during the differentiation phase, which revealed the differentiation potential of single cells. In the presence of FGF4, the proportion of PrE-like cells was higher than upon differentiation in N2B27 alone, and significantly increased with doxycycline induction time between most of the conditions (*P*<0.05, except for 1 h versus 2 h and 4 h versus 8 h; Fig. S2C). Thus, in the presence of exogenous signals, GATA4-mCherry levels control cell-type proportions, in line with previous findings (Schröter et al., 2015).

To separate the effects of extended GATA4-mCherry expression from dosage effects, we analyzed cell differentiation in four clonal cell lines with independent integrations of the GATA4-mCherry transgene. GATA4-mCherry expression levels following 8 h of doxycycline induction varied widely between these lines, both at the population level as well as in single cells (Fig. S3A,B). Yet, in three out of the four clones, similar proportions of PrE-like cells differentiated upon doxycycline removal and culture in N2B27 (*P*<0.05, except for C2 versus all other clones; Fig. S3C,D). When we added recombinant FGF4 during the differentiation phase, the fraction of PrE-like cells was higher than upon differentiation in N2B27 alone, and systematically increased with GATA4-mCherry induction levels (*P*<0.005, ANOVA test for linear trend). In the clonal line with the highest GATA4-mCherry levels, the proportion of PrE-like cells reached a maximum of 98.8±2.0% upon addition of exogenous FGF4 (Fig. S3D), demonstrating that almost all cells have PrE-like differentiation potential following sufficiently strong GATA4-mCherry expression. Taken together, these results indicate that, in minimal medium, the robust proportioning of cell types is established at the population level through cell-cell signaling.

### Differentiating ESCs communicate via FGF4

To identify candidate mechanisms for cell-cell communication that could underlie this robust cell-type proportioning, we focused on FGF4, given that FGF/ERK signaling is required for PrE differentiation both in ESCs and in the embryo (Kang et al., 2013; Krawchuk et al., 2013; Schröter et al., 2015), and that paracrine FGF4 is the main activator of ERK in ESCs (Kunath et al., 2007).

**Fig. 2.**
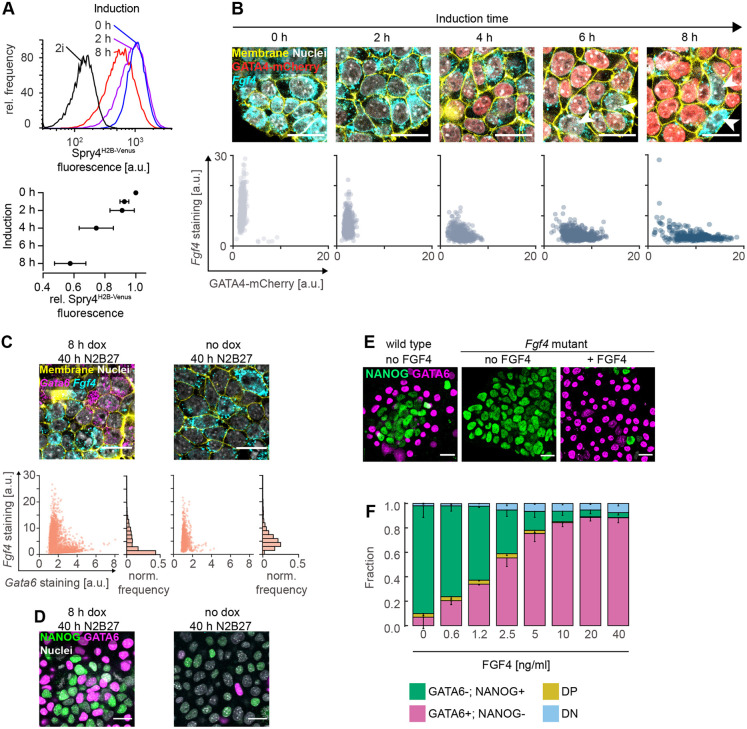
**Differentiating ESCs communicate via FGF4.** (A) Top: flow cytometry histograms showing *Spry4^H2B-Venus^* reporter expression after 24 h of differentiation in N2B27 medium following the indicated durations of doxycycline induction. Black line indicates reporter expression in cells maintained in 2i medium. Bottom: Mean±s.d. of reporter expression from *n*=4 independent experiments, normalized to fluorescence levels of cells transferred to N2B27 without doxycycline induction. (B) Top: GATA4-mCherry protein (red) and *Fgf4* mRNA expression (cyan) in inducible cells at indicated durations of doxycycline induction. Bottom: corresponding single cell quantifications. (C) Top: *Gata6* (magenta) and *Fgf4* mRNA (cyan) expression in inducible cells after 40 h of culture in N2B27 following an 8 h doxycycline pulse (left) or following transfer to N2B27 without induction (right). Bottom: corresponding single cell quantifications. Cell membranes and nuclei in B,C labeled with CellBrite (yellow) and Hoechst 33342 (white), respectively. (D) Immunostaining of cells treated as in C for GATA6 (magenta) and NANOG (green). Nuclei stained with Hoechst 33342 (white). (E) Immunostaining for GATA6 (magenta) and NANOG (green) in wild-type (left) and Fgf4-mutant cells differentiated for 40 h in N2B27 without (middle) or with (right) 10 ng/ml FGF4 after an 8 h doxycycline pulse. (F) Average proportions of cell types in *Fgf4*-mutant cells induced with doxycycline for 8 h, followed by differentiation in N2B27 in the presence of the indicated concentrations of FGF4. GATA6 and NANOG expression were detected by immunostaining and measured by flow cytometry (see Fig. S5). *n*=4; the fraction of GATA6+; NANOG− cells is in magenta, GATA6−; NANOG+ cells is in green, double-positive cells (DP) is in yellow, and double-negative cells (DN) is in blue. Error bars are 95% confidence intervals. Scale bars: 20 µm in B-E.

To investigate how GATA4-mCherry induction levels affect FGF4 signaling, we integrated a *Sprouty4^H2B-Venus^* transcriptional reporter as a quantitative readout for long-term FGF4 signaling (Morgani et al., 2018) in the inducible cell lines. Longer doxycycline induction times corresponding to higher GATA4-mCherry expression levels resulted in reduced mean reporter fluorescence after 24 h of differentiation (Fig. 2A). To test whether this negative correlation between GATA4-mCherry levels and FGF4 signaling was caused by direct regulation of *Fgf4* transcription through GATA factors or via indirect regulation through NANOG (Frankenberg et al., 2011), we used *in situ* mRNA staining for *Fgf4* to determine its expression dynamics following GATA4-mCherry induction. *Fgf4* mRNA was strongly expressed in most cells before induction in 2i medium, but its levels started to decline within 2 h after the start of doxycycline induction (Fig. 2B, left). After 8 h, *Fgf4* mRNA levels were strongly reduced, except in cells with low GATA4-mCherry expression levels (arrowheads in Fig. 2B). Given that most cells still expressed NANOG at this time (Fig. S1A), the rapid downregulation of *Fgf4* mRNA suggests a direct transcriptional regulation through GATA4-mCherry. After 40 h of differentiation in N2B27, *Fgf4* mRNA expression was mutually exclusive with *Gata6* mRNA in cell cultures that had received an 8 h doxycycline pulse (Fig. 2C, left). Without a prior doxycycline pulse, *Fgf4* mRNA continued to be expressed in the majority of cells after 40 h of culture in N2B27 (Fig. 2C, right), although NANOG protein was almost completely downregulated (Fig. 2D). Taken together, these data suggest that GATA factors directly regulate *Fgf4* transcription in ESCs. This possibility was further supported by the presence of a GATA6-binding peak approximately 10 kb upstream of the *Fgf4* start codon in a published ChIP-seq dataset (Wamaitha et al., 2015) that contains a large number of GATA consensus binding sites (Fig. S4A,B). However, in cells in which this putative GATA6-binding site had been deleted, *Fgf4* mRNA expression was downregulated to levels similar to those observed in wild-type cells, both at the end of an 8 h doxycycline pulse, and after 40 h of differentiation (Fig. S4C-E). This suggests that *Fgf4* regulation by GATA factors occurs through multiple, possibly redundant, gene regulatory elements.

Having shown how the cell-intrinsic transcriptional circuits affect FGF4 signaling, we next tested how these circuits were affected by FGF4 dose. To be able to control FGF4 levels, we mutated *Fgf4* in GATA4-mCherry inducible cells. In line with a previous report (Kunath et al., 2007), *Fgf4*-mutant cells continued to express high levels of NANOG upon culture in N2B27, indicative of a failure to initiate epiblast differentiation. This phenotype was rescued to wild-type levels by addition of recombinant FGF4 (Fig. S5A,B). When pulsed for 8 h with doxycycline before culture in N2B27, *Fgf4*-mutant cells likewise continued to express high levels of NANOG, and showed almost no signs of PrE-like differentiation, in contrast to the wild-type control (Fig. 2E,F). PrE-like differentiation was rescued by supplementing recombinant FGF4 during the differentiation phase, resulting in two discrete cell types that had similar NANOG- and GATA6-expression profiles to differentiating wild-type cells (Fig. S5C). The proportions of these cell types depended on FGF4 concentration (Fig. 2E,F; Fig. S5C). Thus, FGF4 signaling and the cell-intrinsic transcriptional circuits underlying cell differentiation mutually regulate each other in a dose-dependent fashion. Therefore, communication via FGF4 is a potential mechanism for coordinating cell differentiation in the population.

### Paracrine FGF4 in ESCs acts locally

**Fig. 3.**
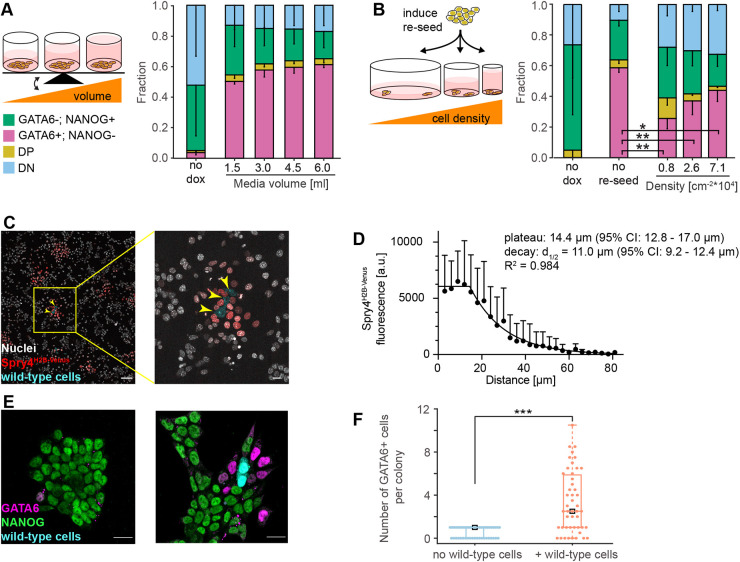
**Communication via FGF4 is spatially restricted.** (A) Left: experimental approach to test effects of media volume on cell-type proportions. Right: average cell-type proportions determined by flow cytometry, *n*=3. (B) Left: experimental approach to test effects of cell density on cell-type proportions. Right: average cell-type proportions determined by flow cytometry, *n*=4; **P<0.005, *P=0.05 (one-way ANOVA). The fraction of GATA6+; NANOG− cells is in magenta, GATA6−; NANOG+ cells is in green, double-positive cells (DP) is in yellow, and double-negative cells (DN) is in blue in A,B. Error bars are 95% confidence intervals. (C) Labeled wild-type cells (cyan, yellow arrowheads) seeded on a layer of *Fgf4*-mutant *Spry4^H2B-Venus^* transcriptional reporter cells. Nuclei are labeled by SiR-Hoechst (white), whereas H2B-Venus fluorescence is in red. (D) Quantitative analysis of FGF4 signaling range. Data points show mean±s.d. of background-subtracted H2B-Venus fluorescence intensities in nuclei of *Spry4^H2B-Venus^* reporter cells per distance bin. Data from *n*=9 independent signaling centers. The fluorescence decay length was estimated by fitting a plateau followed by one-phase exponential decay to the data (black line). (E) Immunostaining for GATA6 (magenta) and NANOG (green) in chimeric cultures 16 h after an 8 h doxycycline pulse and addition of wild-type cells. Staining for GFP (cyan) distinguishes wild-type from *Fgf4*-mutant-inducible cells. One representative image of colonies without (left) or with wild-type cells (right) is shown. (F) Quantification of GATA6-positive cells in colonies without or with wild-type cells, *n*=21 and *n*=25 colonies without and with wild-type cells, respectively. ***P<0.005 (two-tailed Student's *t*-test with unequal variance). Scale bars: 100 µm in C (left) and 20 µm in C (right), E.

We next sought to determine the spatial range of FGF4 signaling in ESCs. We first tested the role of global communication by comparing differentiation outcomes at different medium-to-cell ratios (Fig. 3A). If FGF4 acted globally, ligand concentration would equilibrate in the medium, such that larger volumes would effectively reduce FGF4 concentration, and decrease the proportion of PrE-like cells. In contrast to this expectation, cell-type proportions changed negligibly with media volume (Fig. 3A), indicating that dilution of FGF4 ligands in the medium does not strongly affect cell-type proportioning.

In contrast, to test whether the communication is governed by local FGF4 signaling, we disrupted cell-cell contacts by replating cells at different densities immediately after doxycycline induction (Fig. 3B). Replating strongly reduced the proportion of PrE-like cells compared with the non-trypsinized control (*P*<0.05, Dunnett's multiple comparison test, compare the second to the three rightmost columns in Fig. 3B), indicating that cell-cell contacts in intact colonies support PrE-like differentiation. Furthermore, the proportion of PrE-like cells systematically increased with cell density (three rightmost columns in Fig. 3B, *P*<0.05, one-way ANOVA test for linear trend). These data suggest that cell-cell communication via FGF4 occurs locally and is positively influenced by cell-cell contacts.

To measure the spatial range of FGF4 signaling in ESC colonies directly, we seeded a low number of labeled wild-type cells onto a layer of *Fgf4*-mutant *Spry4^H2B-Venus^* reporter cells (Morgani et al., 2018). After 12 h, H2B-Venus was strongly expressed in a halo of reporter cells immediately surrounding the signal-emitting cells, but reporter expression dropped substantially further away from the sender cells (Fig. 3C). The spatial profile of the H2B-Venus signal was well approximated by a plateau of ∼14.4 µm, followed by an exponential decay with a decay length of ∼11 µm (Fig. 3D). This is likely an overestimate of the immediate effective range of paracrine FGF4 signaling, because the transcriptional reporter integrates signaling activity over the entire duration of the experiment, during which cell divisions and movement will increase the distance between signal-sending and -receiving cells. Delaunay triangulation revealed that, 16 h after the initiation of differentiation, the mean distance between a cell and its nearest and second-nearest neighbors was 14.0±3.2 µm and 25.5±5.3 µm, respectively (Materials and Methods; Fig. S6). Thus, the range of cell-cell communication via FGF4 is spatially restricted and mainly couples nearest and second-nearest neighbors.

We further confirmed the spatially restricted activity of FGF4 using cell differentiation as a readout. When we added a low number of labeled wild-type cells to a culture of *Fgf4*-mutant GATA4-mCherry inducible cells immediately after the end of a doxycycline pulse, PrE-like cells were almost exclusively found in colonies containing *Fgf4* wild-type cells and often localized close to the *Fgf4* sender cells 16 h later (Fig. 3E,F).

### Cell-cell communication via FGF4 underlies the robustness of cell-type proportions

**Fig. 4.**
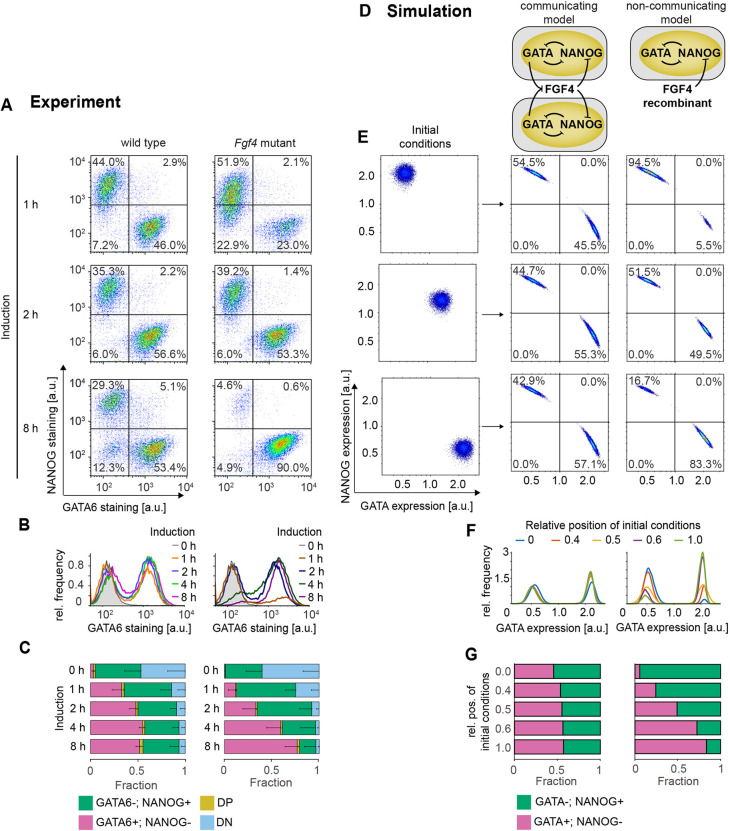
**Cell-cell communication via FGF4 mediates cell-type proportioning.** (A-C) Cell differentiation in wild-type (left) and *Fgf4*-mutant cells (right) after doxycycline pulses of the indicated duration, followed by 40 h of differentiation in N2B27 alone (wild type) or in N2B27 supplemented with 10 ng/ml FGF4 (*Fgf4* mutant). (A) Flow cytometry profiles of NANOG and GATA6 expression. Lines indicate gates used to assign cell types. (B) Marginal distributions of GATA6 staining in wild-type (left) and *Fgf4*-mutant cells (right). (C) Quantification of average cell-type proportions in wild-type (left) and *Fgf4*-mutant cells (right). The fraction of GATA6+; NANOG− cells is in magenta, GATA6−; NANOG+ cells is in green, double-positive cells (DP) is in yellow, and double-negative cells (DN) is in blue. *n*=4, error bars are 95% confidence intervals. (D) Schematic of the model with (left) and without (right) cell-cell communication. (E-G) Influence of initial conditions (left column in E) on GATA6+; NANOG− and GATA6−; NANOG+ cell-type proportions in the model with (middle column) or without cell-cell communication (right column). (E) Distributions of initial conditions (left) and corresponding results of numerical simulations with (middle) or without cell-cell communication (right). Lines in middle and left columns indicate gates used to assign cell types. (F) Marginal distributions of GATA expression, equivalent to B. (G) Quantification of the cell-type proportions obtained from the numerical simulations described in D. See Materials and Methods for model details and parameters.

We next asked whether local communication via FGF4 was the molecular mechanism underlying cell-type proportioning. We titrated GATA4-mCherry expression levels by varying induction time, and compared the robustness of cell-type proportions between wild-type cells that can sense and secrete FGF4, and communication-deficient *Fgf4*-mutant cells rescued with a fixed dose of recombinant FGF4 (Fig. 4A-C). Although the proportions of Epi- and PrE-like cells remained relatively constant in the wild type (Fig. 4A-C, left column, *P*>0.05, except for the proportion of PrE-like cells for 1 h induction versus all other conditions), they were strongly dependent on induction times in *Fgf4*-mutant cells rescued with 10 ng/ml FGF4 (Fig. 4A-C, right column, *P*<0.05, except for the proportion of Epi-like cells for 1 h versus 2 h and 4 h versus 8 h induction). Rescuing *Fgf4*-mutant cells with lower doses of FGF4 decreased the proportion of PrE-like cells and increased the proportion of Epi-like cells, but, for all FGF4 doses tested, cell-type proportions changed with induction time (Fig. S7A). Thus, cell-type proportions in rescued *Fgf4*-mutant cells strongly depend on initial transcription factor expression levels, in contrast to wild-type cells, which can buffer cell-type proportions over a wide range of starting conditions. The crucial difference between *Fgf4* wild-type and rescued mutant cultures is the connectivity of the cellular network: whereas cell differentiation in wild-type cultures is coupled via FGF4, cells in *Fgf4*-mutant cultures take differentiation decisions largely autonomously. We conclude that communication via FGF4 is the molecular mechanism responsible for robust cell-type proportioning in the population.

To further explore how this population-level behavior of robust proportioning arises from cell-cell communication, we determined the dynamical properties of a communicating cell population with numerical simulations. We developed a mathematical description by considering a previously characterized circuit consisting of mutually repressive interactions between GATA factors and NANOG (Schröter et al., 2015) that communicates among single cells through FGF signaling (Fig. 4D, left). Specifically, we posit that GATA factors repress *Fgf4* expression (Fig. 2), and that FGF signaling represses Nanog expression (Hamilton and Brickman, 2014; Schröter et al., 2015). Cell-cell communication was set between nearest and second-nearest neighbors in a population of *N*=10,000 cells on a 100×100 square grid.

In the simulations, we considered a range of initial conditions, from all cells being GATA positive initially to all cells being NANOG positive initially, to mimic the experimental settings (Fig. 4E, left column). Within the coupled population, a stable proportion of two distinct gene expression patterns [(NANOG+; GATA−) or (NANOG−; GATA+)] was established from all starting conditions (Fig. 4E-G, middle). To demonstrate that this robustness of cell-type proportions is a consequence of coupling via FGF, we compared the results to a model in which we replaced communication with a constant exogenous FGF input. This configuration mirrors the situation in the rescued *Fgf4* mutant and effectively models the single cell behavior in which cell differentiation is exclusively governed by the dynamics of the mutually repressive NANOG-GATA circuit. In this non-communicating model, cell-type proportions strongly depended both on the initial condition distributions (Fig. 4D-G, right) and on FGF4 input strength (Fig. S7B), recapitulating experimental results in the rescued *Fgf4* mutant (Fig. 4A-C; Fig. S7A). Finally, we tested the effect of adding exogenous signal to coupled wild-type cells in order to override cell-cell communication. Both in experiments and simulations, this increased the proportion of (NANOG−; GATA+) cells at the expense of (NANOG+; GATA−) cells (Fig. S2B and Fig. S7C,D). Taken together, this congruence between the theoretical and experimental results indicates that recursive cell-cell communication via FGF signaling is sufficient to recapitulate our observation of robust cell-type proportioning.

To explore the dynamical basis of robust cell-type proportioning, we performed bifurcation analysis of a minimal system of *N*=2 communicating cells. This revealed the presence of an inhomogeneous steady state (IHSS), a new collective dynamical state in the coupled system (Fig. S8). An IHSS is composed of mutually exclusive gene expression patterns, is generated and dynamically maintained via communication at the population level and has recently been proposed as a generic mechanism for robust cell-type proportioning (Stanoev et al., 2021). The emergence of an IHSS is therefore a possible dynamical basis for robust cell-type proportioning in differentiating ESCs.

### Spatial organization of cell types indicates a shift from FGF4-dependent to FGF4-independent patterning mechanisms

**Fig. 5.**
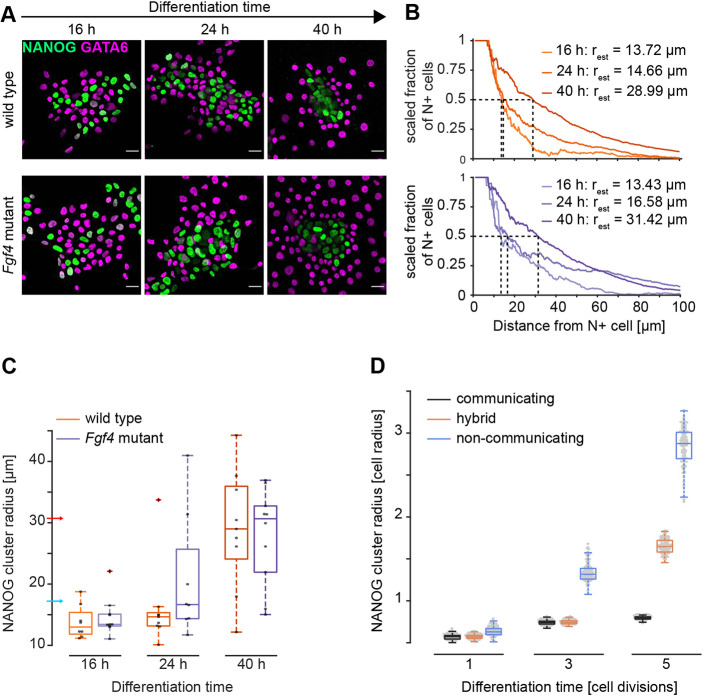
**Spatial arrangement of cell types in wild-type and *Fgf4***-**mutant cells.** (A) Representative immunostainings of wild-type and *Fgf4*-mutant cells for NANOG (green) and GATA6 (magenta) at different time points after the initiation of differentiation. Wild-type cells were induced with doxycycline for 8 h and differentiated in N2B27; *Fgf4*-mutant cells were induced for 4 h and differentiated in N2B27 supplemented with 10 ng/ml FGF4. (B) Estimation of NANOG cluster radius in a single field of view for wild-type (orange, top) and *Fgf4*-mutant cells (blue, bottom) differentiated as in A. Graphs depict the scaled fraction of NANOG+ cells within a specific radius around seed cells. Dashed lines indicate determination of cluster radii. (C) Summary statistics of cluster radii for wild-type (orange) and *Fgf4*-mutant cells (blue) differentiated as in A. Dots indicate values from individual fields of view, box plots show median (boxes), interquartile ranges (whiskers) and outliers (red cross). Blue and red arrows indicate mean distance between nearest and second-nearest neighbors, respectively (Fig. S5). *n*=2, *n*≥8 for wild-type, and *n*=1, *n*≥8 for *Fgf4*-mutant cells. (D) NANOG+ cluster radii quantified from 100 independent numerical realizations of the model with continuous communication (gray), without communication (blue), and of a hybrid model in which communication is switched off after the third division (orange). Box plots show median (boxes) and interquartile ranges (whiskers). Scale bars: 20 µm.

The short spatial range of FGF4 signals in ESC cultures suggests that communication via FGF4 not only leads to robust global cell-type proportions, but also that the differentiated cell types should be arranged in spatial patterns with local structure. Therefore, we analyzed the spatial arrangement of cell types at different time points after the initiation of differentiation. Staining for GATA6 and NANOG indicated that cell types were well mixed after 16 h and 24 h of differentiation, but clustering, particularly of GATA6−; NANOG+ Epi-like cells, was observed after 40 h (Fig. 5A). For further analysis, we focused exclusively on GATA6+; NANOG− (G+) and GATA6−; NANOG+ (N+) cells that we identified with a Gaussian mixture model (Materials and Methods). Analysis of the cell-type composition of the immediate neighborhood N+ and G+ cells corroborated a transition from well-mixed to clustered patterns (Fig. S9A).

To quantify cluster sizes during the differentiation time course, we computed the scaled fraction of N+ cells in neighborhoods of increasing radius around all N+ cells in a field of view (Fig. 5B; Materials and Methods). The value of the scaled fraction is 1 as long as all cells in the neighborhood are N+, and approach zero when the composition of the local neighborhood equals the global composition of cell types. We defined the cluster radius as the distance around N+ cells at which this scaled fraction drops to 0.5 (dashed lines in Fig. 5B). The measure of the cluster radius allows quantification of the length scales of spatially irregular features; however, its absolute value is lower than the physical size of the spatial features. In wild-type cells, the median cluster radius was 13.0 µm and 14.7 µm at 16 h and 24 h of differentiation, respectively, and increased to 29.0 µm at 40 h of differentiation, corresponding to approximately 0.9, 1.0, and 2.1 cell diameters, respectively (Fig. 5C, orange).

To investigate whether these experimentally determined wild-type cluster patterns were consistent with short-range signaling, we quantified the spatial organization of cell types in model simulations. We modeled cluster formation resulting from cell division by considering a dividing cell population, in which the grid size was doubled at each cell division event, and the daughter cells inherited gene expression states from the mother cell (Fig. S10A). In the wild-type case, the cluster radius increased only slightly, from 0.6 to 0.8 cell diameters over five divisions (Fig. 5D; Fig. S10B, gray). Despite the state propagations during the cell divisions, this constant cluster size was maintained by the short-range communication that induces cell-type transitions following each division in the simulation. Thus, the cluster radius in simulations is broadly consistent with the experimentally measured values at early, but not late, stages of differentiation.

Cluster formation at later stages of differentiation could be driven by long-range communication via FGF4, or by FGF4-independent mechanisms. To distinguish between these possibilities, we analyzed the spatial arrangement of cell types in rescued *Fgf4*-mutant cells, using 4 h or 8 h of GATA4-mCherry induction together with differentiation in the presence of 10 ng/ml or 2.5 ng/ml FGF4, respectively, to obtain similar cell-type proportions as in the wild type (Fig. 2B and Fig. 4C). In both *Fgf4*-mutant conditions, cell types were initially well mixed, and clustered at later stages of differentiation (Fig. 5A; Fig. S9B-D). Both the median cluster radius and its increase between 16 h and 40 h were similar in wild-type and in *Fgf4*-mutant cells (Fig. 5B,C, blue; Fig. S11). The continuous increase in cluster size was also seen in simulations of the mutant case. In these simulations, cluster sizes were initially comparable to the wild-type case, but increased rapidly as cells divided, because in the absence of coupling-dependent cell-type transitions, gene expression states and, thus, cell types propagated locally (Fig. 5D; Fig. S10, blue). Consequently, we could recapitulate the transition from a well-mixed to a clustered cell type arrangement in the simulations by removing communication from the system after two cell divisions (Fig. 5D; Fig. S10, orange).

Taken together, these results suggest that, during early stages, the spatial organization of cell types is consistent with regulation by a local cell-cell communication mechanism. However, later on, additional FGF4-independent mechanisms, such as cell division and active cell sorting, dominate the spatial organization.

### Heterogeneous differentiated cell types are maintained by intercellular communication

**Fig. 6.**
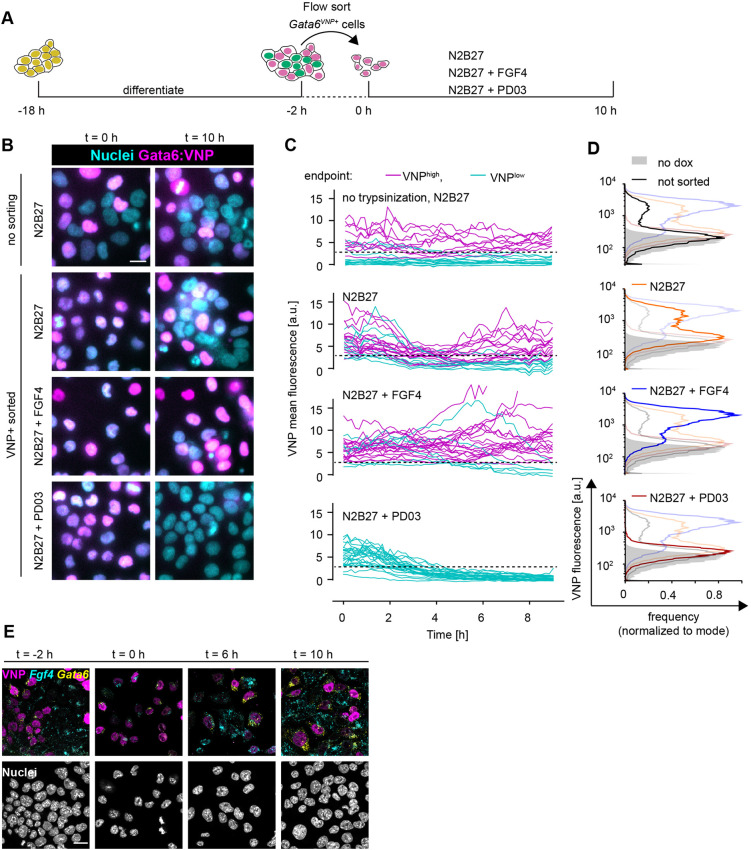
**Heterogeneous cell identities are re-established by cell-cell communication.** (A) Schematic of the cell-sorting experimental protocol. (B) Representative images of Gata6^VNP^ reporter expression in live cells of a non-trypsinized control (upper row) and in cells sorted for VNP expression. Left column is immediately after sorting, right column is after 10 h of culturing in N2B27 medium with the indicated supplements. (C) VNP expression dynamics in individual cells from non-trypsinized colonies (upper panel), or in cells sorted for VNP expression upon culture in the indicated media (lower three panels). Traces are color coded according to expression levels at the end of the experiment (VNP-high, magenta; VNP-low, cyan). Dashed line indicates the threshold used to separate VNP-high from VNP-low cells. (D) Flow cytometry histograms of VNP expression of cells that had not been trypsinized and sorted (black, top), and of cells that had been sorted for VNP expression followed by 10 h of culture in the indicated media. Each panel shows the histogram of the relevant condition as a dark line, and distributions of all other conditions shaded for comparison. Non-induced controls are in gray in all panels. (E) Staining for *Fgf4* (cyan) and *Gata6* (yellow) mRNA in *Gata6^VNP^* reporter cells before sorting (left) and at 2 h, 6 h and 10 h after flow sorting of VNP-positive cells and culture in N2B27. VNP fluorescence is in magenta. Scale bars: 20 µm.

A central characteristic of a population-based mechanism for cell differentiation, such as the IHSS, is the interdependence of different cell types (Koseska and Bastiaens, 2017). It has been demonstrated theoretically that this property of the IHSS solution manifests in the regeneration of heterogeneous populations following separation of cell types after differentiation (Stanoev et al., 2021). To test whether a similar behavior could be observed in isolated PrE-like cells, we used a short-lived Venus-NLS-PEST reporter (Abranches et al., 2013; Nagoshi et al., 2004) knocked into the *Gata6* locus of a GATA4-mCherry inducible cell line as a proxy to isolate PrE-like cells, and subsequently to monitor their differentiation state (Fig. 6A). When putative PrE-like cells were isolated by flow sorting for VNP expression 16 h after the end of a doxycycline pulse and cultured in N2B27 medium, they regenerated a mixture of VNP-positive and -negative cells within 10 h, resembling cell colonies that had not been disrupted and sorted (Fig. 6B-D, first and second rows). The smaller proportion of VNP-positive cells detected by flow cytometry in the unperturbed cultures is likely the result of insufficient induction of the GATA4-mCherry transgene in this cell line (Fig. 6D). Whereas VNP expression was stable over time in most cells in unperturbed colonies, in sorted VNP-positive cells growing in N2B27, the reporter was first globally downregulated before the heterogeneous expression patterns emerged (Fig. 6C, first and second panels, Movies 1 and 2). Similar transitions have been predicted *in silico* as a generic feature of the IHSS solution (Stanoev et al., 2021). This transient downregulation of VNP expression was not observed when the culture medium was supplemented with FGF4, but reporter expression was maintained in the majority of cells (Fig. 6B-D, third row, Movie 3). In contrast, inhibition of FGF/ERK signaling with the MEK inhibitor PD03 resulted in the rapid downregulation of VNP expression following sorting in all cells (Fig. 6B-D, fourth row, Movie 4). Similarly, reporter expression was downregulated in sorted VNP-positive *Fgf4*-mutant cells upon culture in N2B27 alone (Fig. S12). These data indicate that cell-cell communication via FGF4/ERK regulates the re-establishment of a mixture of heterogeneous cell types in a population. *Fgf4* mRNA expression dynamics in sorted VNP-positive cells further supported this idea (Fig. 6E). There was little, if any, detection of *Fgf4* transcripts immediately after sorting, consistent with the repression of *Fgf4* by endogenous GATA6 in VNP-positive cells. However, 6 h later, when the VNP reporter and, hence, endogenous GATA6 expression had decreased, some cells started to re-express *Fgf4* transcripts. A subset of cells re-expressed *Gata6* mRNA and VNP 10 h after sorting, leading to the mutually exclusive expression of VNP, *Fgf4* and *Gata6* mRNA similar to the situation before sorting (Fig. 6E). Taken together, these results indicate that FGF/ERK signaling re-establishes populations with different cell types following the isolation of PrE-like cells. Therefore, in unperturbed cell colonies, intercellular communication via FGF/ERK not only generates, but also actively maintains balanced proportions of differentiated cell types.

## DISCUSSION

Here, we report emergent population-level behavior during the differentiation of Epi- and PrE-like cells from ESCs expressing inducible GATA factors: robust proportions of the two cell types are specified from a wide range of GATA induction levels and re-established from isolated PrE-like cells. This collective behavior relies on local cell-cell communication via FGF4. The observed differentiation characteristics recapitulate the properties of a population-based dynamical solution, an inhomogeneous steady state, recently proposed as a generic mechanism underlying robust differentiation (Stanoev et al., 2021). Our results suggest a new function for FGF signaling, which is to generate and maintain robust proportions of differentiated cell types.

In contrast to previous studies, which reported PrE-like differentiation in *Fgf4*-mutant ESCs upon permanent high-level expression of exogenous GATA factors (Kang et al., 2013; Wamaitha et al., 2015), we found that PrE-like cells do not differentiate from *Fgf4*-mutant ESCs upon transient GATA induction. This recapitulates the *Fgf4*-mutant phenotype in the embryo (Feldman et al., 1995; Kang et al., 2013; Krawchuk et al., 2013). In both *Fgf4*-mutant embryos and ESCs, cell-type proportions can be controlled by recombinant FGF4 in a dose-dependent manner (Krawchuk et al., 2013; Yamanaka et al., 2010). Furthermore, the differentiation of Epi- and PrE-like cells *in vitro* recapitulates the remarkably constant proportions of cell types seen in the developing embryo (Saiz et al., 2016). Lastly, cell identities in ESC populations are plastic and can be respecified upon changing the environment of a cell, again similar to observations in the embryo (Arias et al., 2013; Grabarek et al., 2012). Although the specification of PrE-like cells in ESCs does not occur spontaneously and requires the use of inducible transgenic GATA factors, the parallels between proportioning of Epi- and PrE-like cells in ESCs and the patterning of the ICM of the mouse preimplantation embryo suggest that similar mechanisms operate in both systems. Consistent with this idea, a recent study using chimaeras and targeted ablation of specific cell types concluded that an FGF4-based population-level mechanism balances the size of the Epi and the PrE lineages in the mouse embryo (Saiz et al., 2020). In ESCs, this population-level mechanism manifests in defined media without extrinsic growth factors, in contrast to previously used culture conditions that supply exogenous signals and thereby reveal the dynamics of isolated cell-intrinsic regulatory circuits (Schröter et al., 2015). Thus, the robust proportioning of Epi- and PrE-like cells in defined minimal medium is another example of how endogenous signaling interactions lead to robust patterning of ESC populations (Turner et al., 2017).

The ESC system allowed the identification of new regulatory links of the communication mechanism and the testing of functional properties that had previously been inaccessible. First, our data suggest that the direct repression of *Fgf4* by GATA factors communicates the cell state to the population through a reduction in FGF4 signaling. In ESCs, this new regulatory link appears to predominate over previously proposed mechanisms, such as the indirect regulation of *Fgf4* expression through NANOG, or the regulation of *Fgfr* expression by GATA factors (Frankenberg et al., 2011; Wamaitha et al., 2015). Second, our ability to control transgenic GATA levels experimentally in ESCs allowed us to show that cell-cell communication buffers cell-type proportions against a broad range of initial conditions, as predicted by theory. Third, chimeric cultures of wild-type and *Fgf4*-mutant cells revealed that cell-cell communication via FGF4 signals acts over a short spatial range. This finding suggests a mechanistic explanation for the spatially random differentiation of Epi and PrE cells in the ICM (Chazaud et al., 2006; Fischer et al., 2020; Plusa et al., 2008; Rossant et al., 2003). The short activity range of FGF4 requires cells of opposite fates to be closely juxtaposed until cell fates become irreversibly determined, such that the formation of spatially segregated tissues needs to be achieved through a subsequent sorting step. Our analysis of spatial patterns in ESC cultures indicates that this sequence of first deploying communication via FGF4 to establish spatially intermingled robust proportions of cell types, followed by an FGF-independent sorting step, is conserved *in vitro*.

The repressive coupling of cell fates via short-range FGF4 during ESC differentiation parallels central features of Delta-Notch signaling during lateral inhibition (Ferrell, 2012; Henrique and Schweisguth, 2019; Hori et al., 2013; Simpson, 1990). Consequently, hallmarks of the population-level behavior mediated by FGF4 in differentiating ESCs are recapitulated in an engineered cell system in which cells communicate via Delta-Notch, such as the differentiation of discrete cell types in reproducible proportions, the re-establishment of those proportions upon removal of one cell type, and the dependence of cell-type proportions on cell density or contact (Matsuda et al., 2015). Thus, when connected to appropriate intracellular regulatory circuits, molecularly diverse intercellular communication systems can yield similar functional outputs.

In rescued *Fgf4*-mutant cells that do not communicate, differentiation outcomes in the cell population strongly depend on the distribution of GATA4-mCherry induction levels. This is in line with predictions from single cell models for cell differentiation, in which initial conditions in individual cells strongly influence their differentiation path (Huang et al., 2007). By contrast, when cells communicate via FGF4, the collective differentiation outcome is robust and becomes insensitive to the distribution of GATA4-mCherry induction levels. Thus, the behavior of the communicating cell population cannot be extrapolated directly from the behavior of single isolated cells. Theoretically, the conceptual differences between single cell- and population-based modes of differentiation manifest in the emergence of a new type of solution, an IHSS, that jointly describes the heterogeneous cell identities in the communicating cell population. The robust generation of cell-type proportions irrespective of initial conditions, and their active maintenance through intercellular communication that we observe experimentally, are two key properties of the IHSS (Stanoev et al., 2021). This suggests that the IHSS is a likely dynamical mechanism underlying the differentiation of cells with discrete identities during mammalian preimplantation development. Given the pervasiveness of robust cell-type proportioning during development and homeostasis (Viader-Llargués et al., 2018), it is likely that similar population-based mechanisms underlie canalized development in diverse systems in which multipotent progenitor cells give rise to several differentiated cell types.

## MATERIALS AND METHODS

### Cell lines

Cell lines used in this study were E14tg2a (Hooper et al., 1987) and an *Fgf4*-mutant *Spry4^H2B-Venus/+^* line that we have described previously (Morgani et al., 2018). dsRed-labeled cells were from an E14tg2a-background and kindly supplied by J. Nichols (Department of Physiology, Development and Neuroscience, University of Cambridge, UK). The *Gata6^VNP^* allele reporter was established in the background of a previously described cell line carrying a doxycycline-inducible GATA4-mCherry transgene in the Col1a1 locus as well as a randomly integrated H2B-Cerulean nuclear marker driven by a CAGS promoter (Schröter et al., 2015).

E14tg2a-based inducible cell lines were maintained on fibronectin-coated tissue culture plastic in 2i+LIF medium. The N2B27 basal medium for 2i+LIF was prepared as a 1:1 mixture of DMEM/F12 (PAN Biotech) and Neuropan basal medium (PAN Biotech), supplemented with 0.5% bovine serum albumin, 1× N2 and 1× B27 supplements and 50 µM β-mercaptoethanol (all from Thermo Fisher Scientific). 2i+LIF is N2B27 supplemented with 3 µM CHIR99021 (Tocris), 1 µM PD0325901 (SelleckChem) and 10 ng/ml LIF (Protein Expression Facility, MPI Dortmund). For maintenance of *Fgf4*-mutant subclones, we supplemented the 2i+LIF medium with 10% fetal bovine serum (FBS, Sigma-Aldrich), because *Fgf4*-mutant lines showed severely decreased proliferation upon long-term culture in 2i+LIF alone. FBS was removed at least 1 day before the experiment.

*Spry4* reporter cell lines to measure signaling range, and *Gata6* reporter cell lines were maintained on gelatin-coated dishes in GMEM-based medium supplemented with 10% FBS, sodium pyruvate, 50 µM β-mercaptoethanol, GlutaMAX, non-essential amino acids (all from Thermo Fisher Scientific) and 10 ng/ml LIF. Three days before the experiment, 1 µM PD0325901 was added to the cultures of *Spry4* and *Gata6* reporters to downregulate *Spry4* reporter expression or to capacitate cells for PrE-like differentiation (Schröter et al., 2015).

FGF4 was from Peprotech and supplied in the indicated concentrations, together with 1 µg/ml heparin (Sigma).

### Genetic engineering of ESC lines

Doxycycline-inducible *GATA4*-mCherry-inducible ESCs were generated by electroporation of 50,000 E14tg2a ESCs with 4 µg pPB-TET-GATA4-mCherry, 4 µg pCAG-rtTA-Neo and 4 µg pCAG-PBase (Wang et al., 2008), followed by G418 selection (400 µg/ml) 1 day after transfection. Transgene expression was induced by adding 500 ng/ml doxycycline to the culture medium. We used flow cytometry 2-8 h after transgene induction to select four out of ten independent clonal lines, which showed a range of transgene expression levels. These clonal lines were maintained under G418 selection to circumvent silencing of the inducible transgene.

Mutagenesis of the *Fgf4* locus was performed as previously described (Morgani et al., 2018). *Fgf4* loss-of-function clones were identified by PCR amplification, cloning and sequencing of a sequence around the *Fgf4* start codon. We either selected clones with a targeted mutation delivered by a single-stranded DNA repair template that we have previously shown to disrupt *Fgf4* function (Morgani et al., 2018) or selected at least two independent clones carrying indels around the start codon that introduced frameshift as well as nonsense mutations. All independent clones with random indels showed indistinguishable behavior in the differentiation assays.

The *Gata6* reporter cell line was generated using previously described knockout first-targeting arms of the European Conditional Mouse Mutagenesis Program (EUCOMM) project (Skarnes et al., 2011), combined with a VNP reporter cassette (Nagoshi et al., 2004) and a neomycin resistance gene driven from a human β-actin promoter. This construct was integrated by homologous recombination into a line carrying a doxycycline-inducible GATA4-mCherry transgene in the *Col1a1* locus as well as a randomly integrated H2B-Cerulean nuclear marker driven by a CAGS promoter described in (Schröter et al., 2015). Clones were screened for correct integration of the reporter construct by long-range PCR spanning the targeting arms.

The targeting construct to generate the *Spry4^H2B-Venus^* allele in GATA4-mCherry-inducible cell lines was based on the one used in (Morgani et al., 2018), except that the puromycin-selectable marker was exchanged for a neomycin cassette. The construct was integrated into ESCs by homologous recombination. To increase targeting efficiency, cells were co-transfected with a plasmid expressing Cas9 and a single guide (sg)RNA that targets a sequence near the 5′ end of the 5′-targeting arm which is present in the endogenous *Spry4* locus, but not in the targeting construct. Neomycin-resistant clones were expanded and screened for correct integration of the reporter construct by long-range PCR spanning the targeting arms.

Deletion of the putative GATA-binding element upstream of *Fgf4* was performed by co-transfecting two plasmids expressing Cas9 and sgRNAs flanking the binding element (FGF4_GATAbind_guide5′-2: 5′-AGGGTCTCTGTTCAGGGACA-3′; FGF4_GATAbind_guide3′-1: 5′-CCACATAAGTACCATAGTAT-3′). Following selection for successful transfection, clonal lines were established and tested via PCR with primers Fgf4_GATAbind_fwd2: 5′-GACAGCAACAGTGGATTCAC-3′ and Fgf4_GATAbind_rev2: 5′-ACCCCAGTCTTCTGCAAGAG-3′ for the presence of a deletion of expected size. Deletion of the binding site was further confirmed by Sanger sequencing of PCR amplicons.

All genetically modified lines were karyotyped using standard procedures (Nagy et al., 2008), and all except one clonal line (C5) were confirmed to have a median chromosome count of *n*=40.

### Immunostaining and image analysis

Immunostaining of adherent cells was performed as previously described (Schröter et al., 2015). Antibodies used were anti-NANOG (Thermo Fisher Scientific, 14-5761-80, final concentration 2.5 µg/ml), anti-GATA6 (rabbit polyclonal, Invitrogen, PA1-104, final concentration 5 µg/ml), anti-GATA6 (goat polyclonal, R&D AF1700, final concentration 1 µg/ml), anti-laminin (Sigma-Aldrich, L9393, final concentration 0.5 µg/ml), anti-SOX17 (R&D AF1924, final concentration 1 µg/ml), and anti-FLAG (Sigma-Aldrich, F1804-200, final concentration 1 µg/ml). Secondary antibodies were from Invitrogen/LifeTech. Images were acquired using a 63× 1.4 N.A. oil-immersion objective on a confocal Leica SP8 microscope, with all settings held constant between replicates. Images were quantified using custom scripts written for ImageJ (NIH) and in MATLAB (The MathWorks).

### Flow cytometry

Staining for flow cytometric analysis of intracellular antigens was performed as previously described (Schröter et al., 2015). Primary and secondary antibodies were the same as used for immunostaining. mCherry fluorescence measurements and cell sorting were performed on a BD FACS Aria (BD Biosciences). All other flow cytometric analyses were carried out using a BD LSR II (BD Biosciences). Single cell events were gated based on forward and side scatter properties. GATA4-mCherry expression measurements were normalized to the respective uninduced control. For Fig. S1, cell types were assigned using a Gaussian mixture model (GMM; see below). Otherwise, gates to separate marker-positive from marker-negative cells were determined visually as the threshold that best bisected the bimodal distribution of marker expression across all samples within one experiment.

### Membrane labeling

Cell membranes were labeled with CellBrite Fix (Biotium) according to the manufacturer's recommendations. Briefly, cells were washed with PBS containing Ca and Mg, and then incubated with dye diluted 1:1000 in PBS for 15 min at 37°C. After labeling, cells were washed twice with PBS, followed by fixation and *in situ* hybridization chain reaction (HCR).

### *In situ* HCR and image analysis

Probe sets for *Gata6* and *Fgf4* and corresponding Alexa Fluor-labeled amplifiers for staining of mRNA molecules via third-generation *in situ* HCR (Choi et al., 2018) were sourced from Molecular Instruments. Staining was performed according to the manufacturer's instructions. Briefly, adherent cells were fixed for 15 min with 4% paraformaldehyde, washed with PBS and permeabilized for at least 24 h in 70% ethanol at −20°C. Cells were then washed twice with 2× sodium chloride sodium citrate (SSC) and equilibrated in probe hybridization buffer for at least 30 min. Transcript-specific probes were used at a concentration of 4 nM and hybridized overnight. Excess probe was removed through several washes with probe wash buffer and 5× SSC with 0.1% Tween 20 (SSCT), and cells were equilibrated in amplification buffer for at least 30 min. Fluorescently labeled amplifiers were used at a concentration of 60 nM. Amplification was allowed to proceed for 16-24 h at room temperature. Excess amplifier was removed by several washes with 5× SSCT, followed by counterstaining with Hoechst 33342 (Thermo Fisher Scientific) and mounting in glycerol-based medium. Imaging was performed on an SP8 confocal microscope with a 63× (NA1.4) lens. For image analysis, custom scripts written for ImageJ and MATLAB were used to segment nuclei based on the Hoechst 33342 image, and to identify cells based on the CellBrite membrane stain where available. For images with a cell membrane stain, *Fgf4* mRNA-staining intensities were integrated across the entire cell. For cells differentiated for 40 h, in which identification of the cell outlines with CellBrite proved difficult, we assigned *Fgf4* mRNA signals to individual cells by dilating nuclear masks. Briefly, nuclei were first identified based on the Hoechst 33342 staining, and then dilated using a non-merge dilation. We then measured total mRNA staining within these dilated nuclear masks.

### Analysis of ChIP-seq data

Raw data of GATA6-ChIP-seq from (Wamaitha et al., 2015) were downloaded from NCBI and mapped to the mouse genome (mm10/GRC38) with BowTie2 and default parameters on galaxy.org. Mappings were visualized with Integrative Genomics Viewer (IGV).

### Decay length measurements

*Fgf4*-mutant *Spry4^H2B-Venus^* reporter cells (Morgani et al., 2018) were seeded at a density of 5×10^4^ cells/cm^2^ in N2B27. dsRed-expressing cells were added 2 h later at a density of 500 cells/cm^2^. For the first 3 h of co-culture, the medium was supplemented with 250-500 nM SiR-Hoechst (Lukinavičius et al., 2015) to label nuclei. Live cells were imaged 12 h later on a Leica SP8 confocal microscope. Nuclei were segmented in Fiji (Schindelin et al., 2012) and, for each *Spry4^H2B-Venus^* reporter cell in the vicinity of a dsRed-expressing cell, the background-subtracted Venus fluorescence intensity as well as the distance to the center of mass of the dsRed-expressing cells were determined. Cells were grouped according to their distance from dsRed-expressing cells in 3 µm bins, and mean fluorescence intensities for each bin plotted versus their distance. Decay length was estimated in GraphPad Prism by fitting a plateau followed by a one-phase decay function.

### Cell differentiation in chimeric cultures

*Fgf4*-mutant cells carrying an inducible GATA4-mCherry transgene were seeded at a density of 5×10^4^ cells/cm^2^. Cells were induced 24 h later for 8 h with 500 ng/ml doxycycline in 2i+LIF medium. Medium was switched to N2B27 concomitantly with the addition of 5000 wild-type cells per cm^2^ expressing an ERK-KTR-Clover protein (Raina et al., 2020 preprint) as cell label. Cultures were fixed 16 h later, stained for GATA6, NANOG, GFP and DNA, and both colonies with and without labeled wild-type cells from the same well were imaged on a SP8 confocal microscope. For analysis, we used the blind analysis tool in Fiji. The number of GATA6+;NANOG− cells was scored for each colony without knowing whether the colony contained *Fgf4* wild type, and results were grouped according to the presence or absence of labeled *Fgf4* cells after completion of scoring.

### Determination of cell-cell distances

Neighborhood graphs were constructed for each field of view by using the cell positions to generate a Delaunay triangulation and the corresponding Voronoi diagram. Spurious links between non-adjacent cells were trimmed by excluding links in the Delaunay graph that did not directly pass through the shared Voronoi edge between the two respective Voronoi cells. Links between adjacent cells (purple links in Fig. S6A) were pooled to generate a distance distribution between nearest neighbors (purple histogram, Fig. S6B). The distances of links between unconnected cells that shared a neighbor (yellow in Fig. S6A) were likewise pooled to give the distribution of second-nearest neighbor distances (yellow histogram, Fig. S6B). Both distributions were fit independently with a two-component GMM to separate the true distributions of nearest and second-nearest neighbor distances from higher-order distributions arising from erroneously assigned links (purple- and yellow-dashed lines, Fig. S6B). The mean and s.d. of nearest- and second-nearest neighbor distances were estimated from the first component of these Gaussian fits.

### Assignment of cell types with the Gaussian mixture model

To characterize the emergence of the two main cell types (Fig. S1) and for spatial clustering analysis, we focused on GATA6+; NANOG− (G+) and GATA6−; NANOG+ (N+) cells only. To identify these cell types specifically, we applied a two-component GMM fitted to single cell distributions in the GATA6; NANOG expression space, using the MATLAB function fitgmdist(). The fitted GMM assigns each cell a posterior probability of its association with one of the two component distributions. Cells with a posterior probability of >0.9 for one of the two components were classified to the corresponding cell type. When applying this approach to the flow cytometry data shown in Fig. S1, we also excluded outlier cells. To define outliers for each population of cell types, we first measured the Euclidean distance of each point from its respective population center, defined as the mean of the GMM fit. Next, we eliminated cells with a Euclidean distance of more than four interquartile ranges from the respective population center.

### Analysis of neighborhood composition

To determine local neighborhood composition, we first generated a matrix of Euclidean distances between every cell classified as either G+ or N+ in each field of view. For every cell, we then computed the fraction of G+ and N+ cells within a Euclidean distance of 31.7 µm, encompassing most of the nearest and second-nearest neighbors. Random distributions (blue lines in Fig. S9) were calculated by populating the local neighborhood of each cell randomly from the global distribution in each treatment.

### Measurement of cluster radius

The average cluster radius of N+ cells was estimated with the same method in experimental data in which cell types had been categorized with a GMM, and in simulations of the model. For all N+ cells in a field of view or simulation run, we calculated the decrease in the fraction of N+ cells in neighborhoods of increasing radius, settling to the overall fraction of N+ cells. For scaling, we first subtracted the overall fraction of N+ cells in the respective field of view or simulation run, and then normalized values such that the scaled fraction at zero distance was set to one again. We defined the cluster radius as the distance at which the scaled fraction of N+ cells is equal to 0.5.

### Statistical analysis

Significance testing for cell-type proportions was performed in GraphPad Prism, using one-way ANOVA for matched data with Gaussian distribution, followed by Tukey's or Dunnett's multiple comparison test or a test for a linear trend. In all other conditions, an unpaired, two-tailed Student's *t*-test was used in MATLAB.

### Live cell imaging and tracking

To track *Gata6* reporter expression in live cells, PrE-like differentiation was induced by a 6 h pulse of doxycycline-treatment in serum-containing medium as described in (Schröter et al., 2015). Then, 16 h after doxycycline removal, cells were either switched directly to N2B27 medium lacking Phenol Red or trypsinized, sorted for reporter expression and seeded on fibronectin-coated imaging dishes (ibidi µ-slides). Time-lapse imaging was started within 2 h after sorting on an Olympus IX81 widefield microscope equipped with LED illumination (pE4000, CoolLED) and a Hamamatsu c9100-13 EMCCD camera. Hardware was controlled by MicroManager software (Edelstein et al., 2001). Time-lapse movies were acquired using a 40× oil immersion lens (NA 1.2), with 10 min time intervals.

Cell tracking was carried out with TrackMate (ImageJ) (Tinevez et al., 2017) based on the constitutively expressed H2B-Cerulean nuclear marker. Fluorescence intensity was measured in a circular region of interest in the center of the nucleus, and background-subtracted fluorescence intensities plotted in Python. Trace color in Fig. 6D was assigned according to fluorescence intensity in the last frame of the movie, with respect to the estimated intensity threshold used for flow sorting (dashed line in Fig. 6D).

### Computational model for cell-type proportioning

The model of the intercellular communication system (Fig. 4D) is adapted from (Stanoev et al., 2021), and is described by Eqns 1-3:
(1)

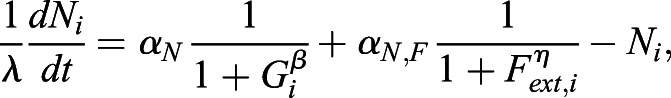

(2)

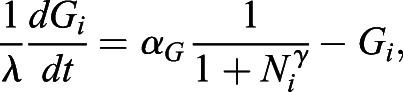

(3)

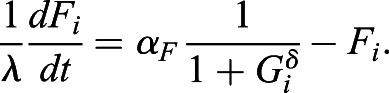
*N*_*i*_ and *G*_*i*_ describe NANOG and GATA6 protein expression levels in cell *i*, regulated by mutual inhibition, whereas *F*_*i*_ is the secreted FGF4 the production of which is downregulated by GATA6. Eqn 4 determines the extracellular FGF4 concentration that is sensed by cell *i* from its neighborhood *N*(*i*), resulting in downregulation of NANOG production in the cell.
(4)

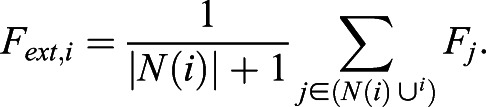
*α*_*N*_=2.5, *α*_*N*,*F*_=0.5, *α*_*G*_=3 and *α*_*F*_=3 denote production rate constants, *β*=*η*=*γ*=*δ*=2 are the Hill coefficients, degradation rates were set to 1 because *λ*=50 was used as a scaling kinetic parameter. In total, 10,000 cells were deployed on a regular 100×100 two-dimensional lattice with no-flux boundary conditions. Cell-cell communication was modeled to be short range, reflecting the experimental wild-type case, (*i.e.* communication between direct neighbors and cells two hops away on the lattice). When mimicking the *Fgf4*-mutant case, communication between cells was excluded, and an external input was modeled with *F*_*ext*_=1.2 for the results shown in Fig. 4E-G. For Fig. S7B, *F*_*ext*_ was varied as indicated. When supplementing external input in the wild-type case, *F*_*ext*_=1.0 was added to communication input *F*_*ext*,*i*_ for each cell *i*.

The cell populations were initiated analogously to the experimental case by varying the initial conditions of all cells from being NANOG expressing, through intermediate NANOG and GATA6 expression, to being GATA6 expressing (Fig. 4E, left column). More specifically, the variables were sampled independently from unimodal Gaussian distributions 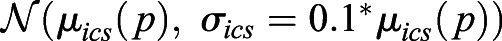, with the mean *μ*_*ics*_(*p*)=(1−*p*)*μ_*G*−;*N*+_+p*μ_*G*+;*N*−_ placed on the line segment connecting the GATA6−; NANOG+ state *μ*_*G*−;*N*+_ and the GATA6+; NANOG- state *μ*_*G*+;*N*−_, partitioning it in proportion *p*. *p*∈{0, 0.4, 0.5, 0.6, 1} was used for the quantifications in Fig. 4G. Samples from around the endpoints and the midpoint 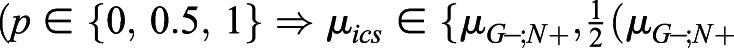 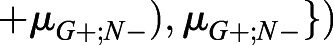 are shown in the left column of Fig. 4E.

Cell heterogeneity was introduced by varying all of the parameters independently with s.d. of 0.02 from the respective values for each cell. A stochastic differential equation model was constructed from the deterministic equations by adding a multiplicative noise term *σXdW*_*t*_, in which *dW*_*t*_ is the Brownian motion term, *X* is the variable state and *σ*=0.1 is the noise term. The model was solved with Δ*t*=0.01 using the Milstein method (Mil'shtejn, 1975). Following integration, cell identities were estimated by comparing the NANOG and GATA6 values from the final states of the cells, and the ratios were computed.

For comparing the spatial organizations between communicating and non-communicating cells at different time points, periodic synchronous cell divisions in the population were included in the model as in (Stanoev et al., 2021), spanning five cell cycles. Cell divisions occur along the horizontal and vertical axes on the grid alternately, sequentially yielding lattices of 10×10, 10×20, 20×20, 20×40 and 40×40 cells (Fig. S10A). At every cell division, the final state of the mother cell is passed on to the initial conditions of the daughter cells. The parameter set of the mother cell is also inherited. Spatial organizations were analyzed at the end of each cell cycle, after the collective state had been allowed to reach a steady state in a deterministic fashion, by estimating the N+ cluster radius as described above.

For the hybrid model, it was assumed that, after the third cell cycle, the cells commit to their current fates and the communication becomes inconsequential, effectively bringing about a switch to a non-communicating grid. For all conditions, cell states were initialized with Gaussians with *μ*_*ics*_(0.5), as described above.

The numerical bifurcation analysis for the two-cell system (Fig. S8) was performed using the XPP/AUTO software (http://www.math.pitt.edu/~bard/xpp/xpp.html). All simulations were performed using custom-made code in MATLAB.

## Supplementary Material

Supplementary information

Reviewer comments

## References

[DEV199926C1] Abranches, E., Bekman, E. and Henrique, D. (2013). Generation and characterization of a novel mouse embryonic stem cell line with a dynamic reporter of nanog expression. *PLoS ONE* 8, e59928. 10.1371/journal.pone.0059928 23527287 PMC3602340

[DEV199926C2] Arias, A. M., Nichols, J. and Schröter, C. (2013). A molecular basis for developmental plasticity in early mammalian embryos. *Development* 140, 3499-3510. 10.1242/dev.091959 23942513

[DEV199926C3] Bedzhov, I., Graham, S. J. L., Leung, C. Y. and Zernicka-Goetz, M. (2014). Developmental plasticity, cell fate specification and morphogenesis in the early mouse embryo. *Philos. Trans. R. Soc. B Biol. Sci.* 369, 20130538. 10.1098/rstb.2013.0538 PMC4216461 25349447

[DEV199926C4] Bessonnard, S., De Mot, L., Gonze, D., Barriol, M., Dennis, C., Goldbeter, A., Dupont, G. and Chazaud, C. (2014). Gata6, Nanog and Erk signaling control cell fate in the inner cell mass through a tristable regulatory network. *Development* 141, 3637-3648. 10.1242/dev.109678 25209243

[DEV199926C5] Bradley, A., Evans, M., Kaufman, M. H. and Robertson, E. (1984). Formation of germ-line chimaeras from embryo-derived teratocarcinoma cell lines. *Nature* 309, 255-256. 10.1038/309255a0 6717601

[DEV199926C6] Chazaud, C., Yamanaka, Y., Pawson, T. and Rossant, J. (2006). Early lineage segregation between epiblast and primitive endoderm in mouse blastocysts through the Grb2-MAPK pathway. *Dev. Cell* 10, 615-624. 10.1016/j.devcel.2006.02.020 16678776

[DEV199926C7] Chickarmane, V. and Peterson, C. (2008). A computational model for understanding stem cell, trophectoderm and endoderm lineage determination. *PLoS ONE* 3, e3478-e3478. 10.1371/journal.pone.0003478 18941526 PMC2566811

[DEV199926C8] Choi, H. M. T., Schwarzkopf, M., Fornace, M. E., Acharya, A., Artavanis, G., Stegmaier, J., Cunha, A. and Pierce, N. A. (2018). Third-generation in situ hybridization chain reaction: multiplexed, quantitative, sensitive, versatile, robust. *Development* 145, dev165753. 10.1242/dev.165753 29945988 PMC6031405

[DEV199926C10] De Caluwé, J., Tosenberger, A., Gonze, D. and Dupont, G. (2019). Signalling-modulated gene regulatory networks in early mammalian development. *J Theor. Biol.* 463, 56-66. 10.1016/j.jtbi.2018.12.008 30543809

[DEV199926C11] De Mot, L., Gonze, D., Bessonnard, S., Chazaud, C., Goldbeter, A. and Dupont, G. (2016). Cell fate specification based on tristability in the inner cell mass of mouse blastocysts. *Biophys. J.* 110, 710-722. 10.1016/j.bpj.2015.12.020 26840735 PMC4744165

[DEV199926C12] Edelstein, A., Amodaj, N., Hoover, K., Vale, R. and Stuurman, N. (2001). *Computer Control of Microscopes Using µManager*. John Wiley & Sons, Inc. 10.1002/0471142727.mb1420s92 PMC3065365 20890901

[DEV199926C13] Feldman, B., Poueymirou, W., Papaioannou, V. E., DeChiara, T. M. and Goldfarb, M. (1995). Requirement of FGF-4 for postimplantation mouse development. *Science* 267, 246-249. 10.1126/science.7809630 7809630

[DEV199926C14] Ferrell, J. E. (2012). Bistability, bifurcations, and waddington's epigenetic landscape. *Curr. Biol.* 22, R458-R466. 10.1016/j.cub.2012.03.045 22677291 PMC3372930

[DEV199926C15] Fischer, S. C., Corujo-Simon, E., Lilao-Garzon, J., Stelzer, E. H. K. and Muñoz-Descalzo, S. (2020). The transition from local to global patterns governs the differentiation of mouse blastocysts. *PLoS ONE* 15, e0233030. 10.1371/journal.pone.0233030 32413083 PMC7228118

[DEV199926C16] Frankenberg, S., Gerbe, F., Bessonnard, S., Belville, C., Pouchin, P., Bardot, O. and Chazaud, C. (2011). Primitive endoderm differentiates via a three-step mechanism involving Nanog and RTK signaling. *Dev. Cell* 21, 1005-1013. 10.1016/j.devcel.2011.10.019 22172669

[DEV199926C17] Gardner, R. L. (1968). Mouse chimaeras obtained by the injection of cells into the blastocyst. *Nature* 220, 596-597. 10.1038/220596a0 5686740

[DEV199926C18] Grabarek, J. B., Żyżyńska, K., Saiz, N., Piliszek, A., Frankenberg, S., Nichols, J., Hadjantonakis, A.-K. and Plusa, B. (2012). Differential plasticity of epiblast and primitive endoderm precursors within the ICM of the early mouse embryo. *Development* 139, 129-139. 10.1242/dev.067702 22096072 PMC3231774

[DEV199926C19] Hamilton, W. B. and Brickman, J. M. (2014). Erk signaling suppresses embryonic stem cell self-renewal to specify endoderm. *Cell Rep.* 9, 2056-2070. 10.1016/j.celrep.2014.11.032 25533345

[DEV199926C20] Henrique, D. and Schweisguth, F. (2019). Mechanisms of Notch signaling: a simple logic deployed in time and space. *Development* 146, dev172148. 10.1242/dev.172148 30709911

[DEV199926C21] Hooper, M., Hardy, K., Handyside, A., Hunter, S. and Monk, M. (1987). HPRT-deficient (Lesch-Nyhan) mouse embryos derived from germline colonization by cultured cells. *Nature* 326, 292-295. 10.1038/326292a0 3821905

[DEV199926C22] Hori, K., Sen, A. and Artavanis-Tsakonas, S. (2013). Notch signaling at a glance. *J. Cell Sci.* 126, 2135-2140. 10.1242/jcs.127308 23729744 PMC3672934

[DEV199926C23] Huang, S., Guo, Y.-P., May, G. and Enver, T. (2007). Bifurcation dynamics in lineage-commitment in bipotent progenitor cells. *Dev. Biol.* 305, 695-713. 10.1016/j.ydbio.2007.02.036 17412320

[DEV199926C24] Kang, M., Piliszek, A., Artus, J. and Hadjantonakis, A.-K. (2013). FGF4 is required for lineage restriction and salt-and-pepper distribution of primitive endoderm factors but not their initial expression in the mouse. *Development* 140, 267-279. 10.1242/dev.084996 23193166 PMC3597205

[DEV199926C25] Kang, M., Garg, V. and Hadjantonakis, A.-K. (2017). Lineage establishment and progression within the inner cell mass of the mouse blastocyst requires FGFR1 and FGFR2. *Dev. Cell* 41, 496-510.e5. 10.1016/j.devcel.2017.05.003 28552559 PMC5530874

[DEV199926C26] Koseska, A. and Bastiaens, P. I. H. (2017). Cell signaling as a cognitive process. *EMBO J.* 36, 568-582. 10.15252/embj.201695383 28137748 PMC5331751

[DEV199926C27] Krawchuk, D., Honma-Yamanaka, N., Anani, S. and Yamanaka, Y. (2013). FGF4 is a limiting factor controlling the proportions of primitive endoderm and epiblast in the ICM of the mouse blastocyst. *Dev. Biol.* 384, 65-71. 10.1016/j.ydbio.2013.09.023 24063807

[DEV199926C28] Kunath, T., Saba-El-Leil, M. K., Almousailleakh, M., Wray, J., Meloche, S. and Smith, A. (2007). FGF stimulation of the Erk1/2 signalling cascade triggers transition of pluripotent embryonic stem cells from self-renewal to lineage commitment. *Development* 134, 2895-2902. 10.1242/dev.02880 17660198

[DEV199926C29] Lukinavičius, G., Blaukopf, C., Pershagen, E., Schena, A., Reymond, L., Derivery, E., Gonzalez-Gaitan, M., D'Este, E., Hell, S. W., Gerlich, D. W. et al. (2015). SiR-Hoechst is a far-red DNA stain for live-cell nanoscopy. *Nat. Commun.* 6, 8497. 10.1038/ncomms9497 26423723 PMC4600740

[DEV199926C30] Matsuda, M., Koga, M., Woltjen, K., Nishida, E. and Ebisuya, M. (2015). Synthetic lateral inhibition governs cell-type bifurcation with robust ratios. *Nat. Commun.* 6, 6195. 10.1038/ncomms7195 25652697

[DEV199926C31] Mil'shtejn, G. N. (1975). Approximate integration of stochastic differential equations. *Theory Probab. Appl.* 19, 557-562. 10.1137/1119062

[DEV199926C32] Molotkov, A., Mazot, P., Brewer, J. R., Cinalli, R. M. and Soriano, P. (2017). Distinct requirements for FGFR1 and FGFR2 in primitive endoderm development and exit from pluripotency. *Dev. Cell* 41, 511-526.e4. 10.1016/j.devcel.2017.05.004 28552557 PMC5502766

[DEV199926C33] Morgani, S. M., Saiz, N., Garg, V., Raina, D., Simon, C. S., Kang, M., Arias, A. M., Nichols, J., Schröter, C. and Hadjantonakis, A.-K. (2018). A Sprouty4 reporter to monitor FGF/ERK signaling activity in ESCs and mice. *Dev. Biol.* 441, 104-126. 10.1016/j.ydbio.2018.06.017 29964027 PMC6455974

[DEV199926C34] Nagoshi, E., Saini, C., Bauer, C., Laroche, T., Naef, F. and Schibler, U. (2004). Circadian gene expression in individual fibroblasts: cell-autonomous and self-sustained oscillators pass time to daughter cells. *Cell* 119, 693-705. 10.1016/j.cell.2004.11.015 15550250

[DEV199926C35] Nagy, A., Gertsenstein, M., Vintersten, K. and Behringer, R. (2008). Karyotyping Mouse Cells. *Cold Spring Harb. Protoc.* 3, pdb.prot4706. 10.1101/pdb.prot4706 21356825

[DEV199926C36] Niakan, K. K., Ji, H., Maehr, R., Vokes, S. A., Rodolfa, K. T., Sherwood, R. I., Yamaki, M., Dimos, J. T., Chen, A. E., Melton, D. A. et al. (2010). Sox17 promotes differentiation in mouse embryonic stem cells by directly regulating extraembryonic gene expression and indirectly antagonizing self-renewal. *Genes Dev.* 24, 312-326. 10.1101/gad.1833510 20123909 PMC2811832

[DEV199926C37] Plusa, B., Piliszek, A., Frankenberg, S., Artus, J. and Hadjantonakis, A.-K. (2008). Distinct sequential cell behaviours direct primitive endoderm formation in the mouse blastocyst. *Development* 135, 3081-3091. 10.1242/dev.021519 18725515 PMC2768606

[DEV199926C38] Raina, D., Fabris, F., Morelli, L. G. and Schröter, C. (2020). Intermittent ERK oscillations downstream of FGF in mouse embryonic stem cells. *bioRxiv* 10.1101/2020.12.14.422687 PMC8918804 35175328

[DEV199926C39] Rossant, J., Chazaud, C. and Yamanaka, Y. (2003). Lineage allocation and asymmetries in the early mouse embryo. *Philos. Trans. R. Soc. B Biol. Sci.* 358, 1341-1349. 10.1098/rstb.2003.1329 PMC1693231 14511480

[DEV199926C40] Saiz, N., Williams, K. M., Seshan, V. E. and Hadjantonakis, A.-K. (2016). Asynchronous fate decisions by single cells collectively ensure consistent lineage composition in the mouse blastocyst. *Nat. Commun.* 7, 13463. 10.1038/ncomms13463 27857135 PMC5120222

[DEV199926C41] Saiz, N., Mora-Bitria, L., Rahman, S., George, H., Herder, J. P., Garcia-Ojalvo, J. and Hadjantonakis, A.-K. (2020). Growth-factor-mediated coupling between lineage size and cell fate choice underlies robustness of mammalian development. *eLife* 9, 6289. 10.7554/eLife.56079 PMC7513828 32720894

[DEV199926C42] Schindelin, J., Arganda-Carreras, I., Frise, E., Kaynig, V., Longair, M., Pietzsch, T., Preibisch, S., Rueden, C., Saalfeld, S., Schmid, B. et al. (2012). Fiji: an open-source platform for biological-image analysis. *Nat. Methods* 9, 676-682. 10.1038/nmeth.2019 22743772 PMC3855844

[DEV199926C43] Schröter, C., Rué, P., Mackenzie, J. P. and Arias, A. M. (2015). FGF/MAPK signaling sets the switching threshold of a bistable circuit controlling cell fate decisions in embryonic stem cells. *Development* 142, 4205-4216. 10.1242/dev.127530 26511924 PMC4689219

[DEV199926C44] Simon, C. S., Hadjantonakis, A.-K. and Schröter, C. (2018). Making lineage decisions with biological noise: lessons from the early mouse embryo. *Wiley Interdiscip. Rev. Dev. Biol.* 7, e319. 10.1002/wdev.319 29709110 PMC6002940

[DEV199926C45] Simpson, P. (1990). Lateral inhibition and the development of the sensory bristles of the adult peripheral nervous system of *Drosophila*. *Development* 109, 509-519. 10.1242/dev.109.3.509 2205467

[DEV199926C46] Skarnes, W. C., Rosen, B., West, A. P., Koutsourakis, M., Bushell, W., Iyer, V., Mujica, A. O., Thomas, M., Harrow, J., Cox, T. et al. (2011). A conditional knockout resource for the genome-wide study of mouse gene function. *Nature* 474, 337-342. 10.1038/nature10163 21677750 PMC3572410

[DEV199926C47] Stanoev, A., Schröter, C. and Koseska, A. (2021). Robustness and timing of cellular differentiation through population-based symmetry breaking. *Development* 148, dev197608. 10.1242/dev.197608 33472845

[DEV199926C48] Tarkowski, A. K. (1959). Experiments on the development of isolated blastomeres of mouse eggs. *Nature* 184, 1286-1287. 10.1038/1841286a0 13836947

[DEV199926C49] Tarkowski, A. K. (1961). Mouse chimæras developed from fused eggs. *Nature* 190, 857-860. 10.1038/190857a0 13775333

[DEV199926C50] Tinevez, J.-Y., Perry, N., Schindelin, J., Hoopes, G. M., Reynolds, G. D., Laplantine, E., Bednarek, S. Y., Shorte, S. L. and Eliceiri, K. W. (2017). TrackMate: an open and extensible platform for single-particle tracking. *Methods* 115, 80-90. 10.1016/j.ymeth.2016.09.016 27713081

[DEV199926C51] Turner, D. A., Girgin, M., Alonso-Crisostomo, L., Trivedi, V., Baillie-Johnson, P., Glodowski, C. R., Hayward, P. C., Collignon, J., Gustavsen, C., Serup, P. et al. (2017). Anteroposterior polarity and elongation in the absence of extra-embryonic tissues and of spatially localised signalling in gastruloids: mammalian embryonic organoids. *Development* 144, 3894-3906. 10.1242/dev.150391 28951435 PMC5702072

[DEV199926C52] Viader-Llargués, O., Lupperger, V., Pola-Morell, L., Marr, C. and López-Schier, H. (2018). Live cell-lineage tracing and machine learning reveal patterns of organ regeneration. *eLife* 7, e08201. 10.7554/eLife.30823 PMC5903862 29595471

[DEV199926C53] Waddington, C. H. (1942). Canalization of development and the inheritance of acquired characters. *Nature* 150, 563-565. 10.1038/150563a0 13666847

[DEV199926C54] Wamaitha, S. E., del Valle, I., Cho, L. T. Y., Wei, Y., Fogarty, N. M. E., Blakeley, P., Sherwood, R. I., Ji, H. and Niakan, K. K. (2015). Gata6 potently initiates reprograming of pluripotent and differentiated cells to extraembryonic endoderm stem cells. *Genes Dev.* 29, 1239-1255. 10.1101/gad.257071.114 26109048 PMC4495396

[DEV199926C55] Wang, W., Lin, C., Lu, D., Ning, Z., Cox, T., Melvin, D., Wang, X., Bradley, A. and Liu, P. (2008). Chromosomal transposition of PiggyBac in mouse embryonic stem cells. *Proc. Natl Acad. Sci. USA* 105, 9290-9295. 10.1073/pnas.0801017105 18579772 PMC2440425

[DEV199926C56] Yamanaka, Y., Lanner, F. and Rossant, J. (2010). FGF signal-dependent segregation of primitive endoderm and epiblast in the mouse blastocyst. *Development* 137, 715-724. 10.1242/dev.043471 20147376

[DEV199926C57] Ying, Q.-L., Wray, J., Nichols, J., Batlle-Morera, L., Doble, B., Woodgett, J., Cohen, P. and Smith, A. (2008). The ground state of embryonic stem cell self-renewal. *Nature* 453, 519-523. 10.1038/nature06968 18497825 PMC5328678

