# Supplementary material for: Cell-cell communication through FGF4 generates and maintains robust proportions of differentiated cell types in embryonic stem cells
Source: Development. 2021 Nov 5;148(21):dev199926. doi: 10.1242/dev.199926 (PMC8602943; doi:10.1242/dev.199926)
Supplement: Supplementary information [file develop-148-199926-s1.pdf]

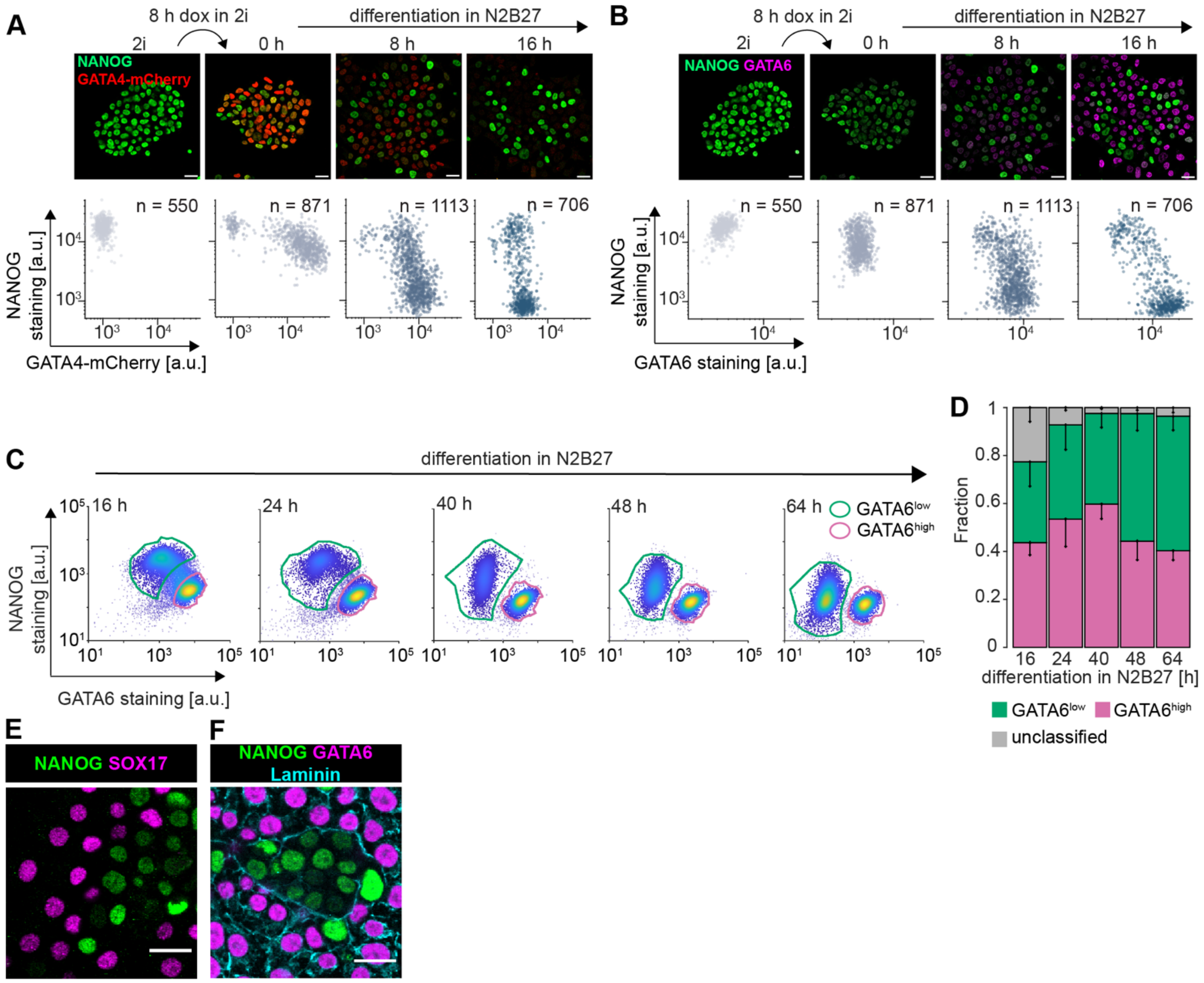

Fig. S1. Differentiation of Epi- and PrE-like cells following pulsed GATA4-mCherry expression.
(A) Immunostaining (top) and single cell quantification (bottom) of GATA4-mCherry (red) and NANOG expression (green) in Gata4-mCherry inducible cells cultured in 2i + LIF medium (left) or at indicated times after the end of an 8 h doxycycline pulse. (B) Same cells as in(A), but showing immunostaining (top) and single cell quantification (bottom) of GATA6 (magenta) and NANOG (green) expression. (C) Flow cytometry profiles of Gata4-mCherry inducible cells stained for NANOG and GATA6 after an 8 h doxycycline pulse, followed by differentiation in N2B27 medium for the indicated durations. Green and magenta lines surround cells that were assigned to one of the two main clusters by a Gaussian mixture model (Materials and Methods). (D) Average proportions of cells assigned to one of the two main clusters shown in **C**. Proportion of GATA6^{high} cells is in magenta, proportion of GATA6^{low} cells is in green, and unassigned cells are in gray. n = 3, error bars are 95% confidence intervals. (E) Immunostaining for SOX17 (magenta) and NANOG (green) in GATA4-mCherry inducible cells cultured for 40 h in N2B27 after an 8 h doxycycline pulse. (F) Immunostaining for GATA6 (magenta), NANOG (green), and Laminin (cyan) in GATA4-mCherry inducible cells cultured for 40 h in N2B27 after an 8 h doxycycline pulse. Scale bars: 20 μm in A, B, E, F.

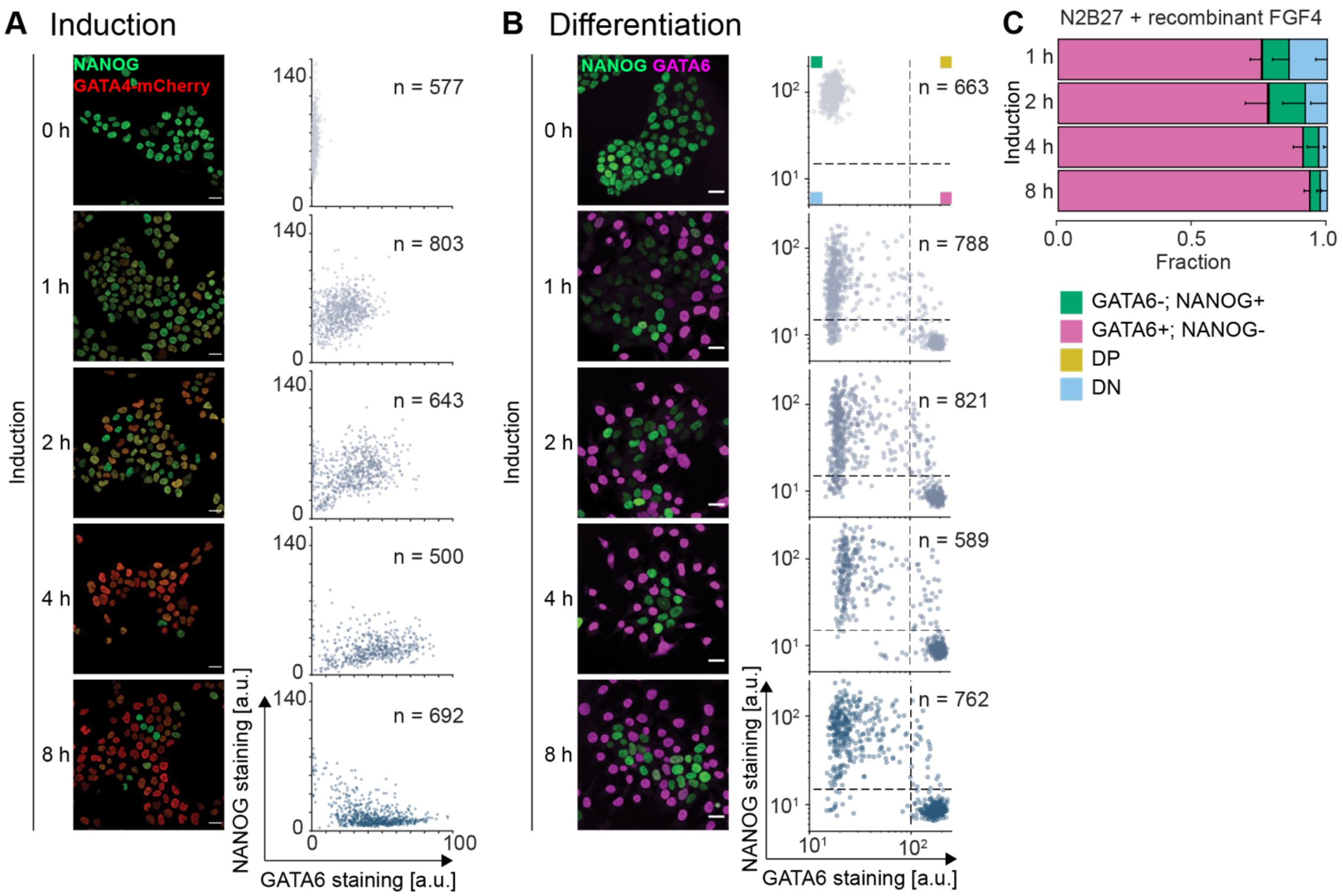

Fig. S2. Quantitative immunofluorescence analysis of NANOG and GATA expression for different doxycycline induction times. (A) Immunostaining (left) and single-cell quantification (right) of NANOG (green) and GATA4-mCherry (red) expression in inducible cells after indicated durations of doxycycline treatment in 2i + LIF medium. (B) Immunostaining (left) and single-cell quantification (right) of NANOG (green) and GATA6 (magenta) expression in inducible cells after indicated durations of doxycycline treatment, followed by 40 h differentiation in N2B27 medium. Immunofluorescence micrographs in A and B for 0, 2, and 8 h of doxycycline induction are reproduced from Fig. 1 for comparison. Dashed lines in scatter plots in B indicate thresholds to assign cell types; upper left quadrant: GATA6-; NANOG+; lower right quadrant GATA6+; NANOG-; upper right quadrant double positive (DP); lower left quadrant double negative (DN). (C) Proportions of cell types upon indicated durations of doxycycline induction followed by 40 h of differentiation in N2B27 medium supplemented with 10 ng/ml FGF4. Cell identities were determined by immunostaining and quantitative immunofluorescence. Fraction of GATA6+, NANOG- cells in magenta, GATA6-, NANOG+ cells in green, double positive cells (DP) in yellow, and double negative cells (DN) in blue. n = 4, error bars indicate 95% confidence intervals. Scale bars: 20 µm in A, B.

Development • Supplementary information

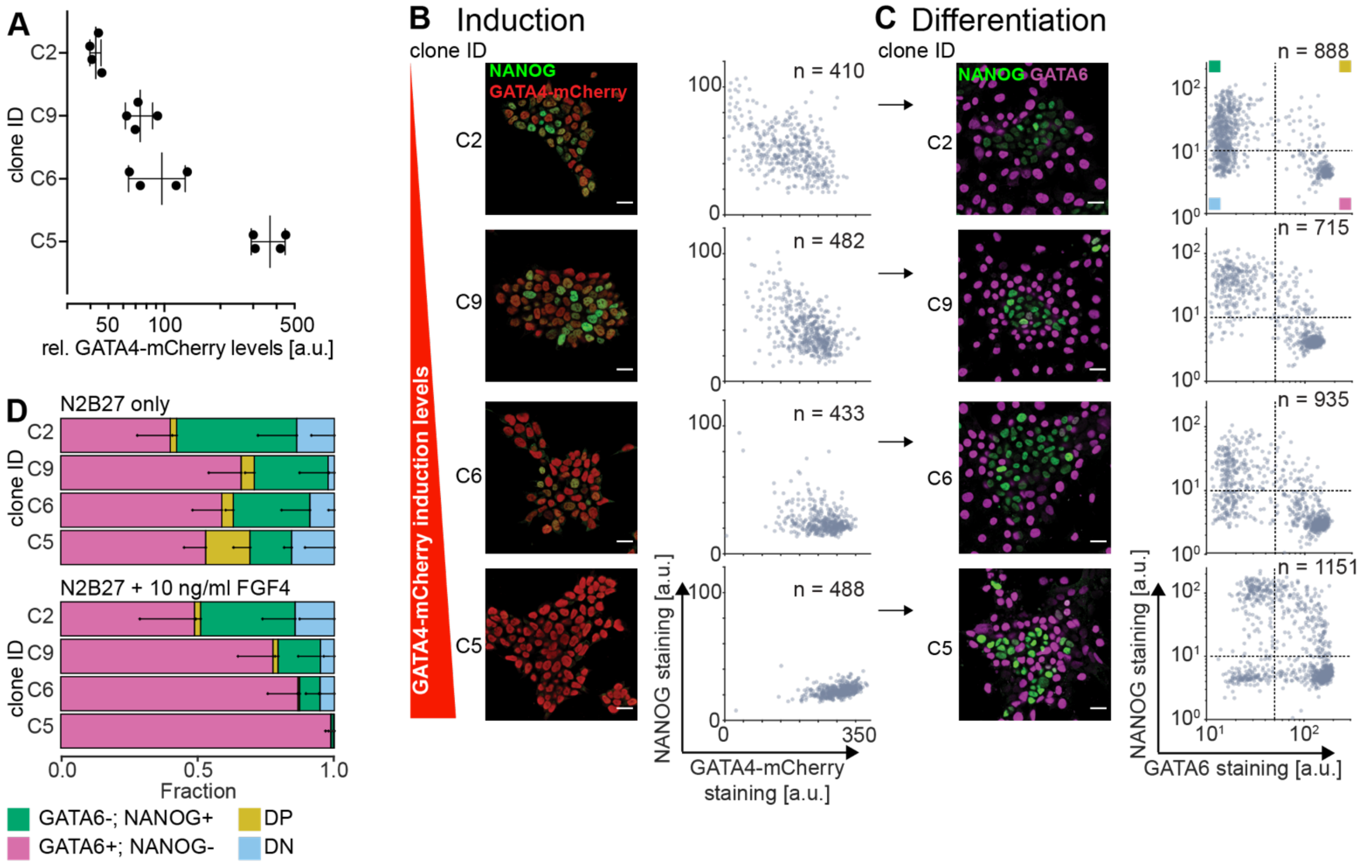

Fig. S3. Robust cell-type proportioning in independent clonal cell lines. (A) GATA4-mCherry induction levels in four independent clonal inducible cell lines after 8 h of doxycycline treatment in 2i + LIF medium, measured by flow cytometry. Clones are ordered by GATA4-mCherry expression strength, fluorescence values were normalized to non-induced control cells. Plot shows individual data points and mean ± s.d. from n = 4 independent experiments. (B) Immunostaining (left) and single-cell quantification (right) of NANOG (green) and GATA4-mCherry (red) expression in the same clonal lines analysed in A after 8 h of doxycycline stimulation in 2i + LIF medium. (C) Immunostaining (left) and single cell quantification (right) of NANOG (green) and GATA6 (magenta) expression in cells from independent clonal lines treated with doxycycline for 8 h and differentiated in N2B27 for 40 h. Dashed lines: Thresholds to determine cell types; upper left quadrant: GATA6-; NANOG+; lower right quadrant GATA6+; NANOG-; upper right quadrant double positive (DP); lower left quadrant double negative (DN). Clones are ordered by GATA4-mCherry induction strength as in A. (D) Average cell-type proportions in clonal lines differentiated as in **C** in N2B27 only (top) or in N2B27 supplemented with 10 ng/ml FGF4 (bottom). Fraction of GATA6+; NANOG- cells in magenta, GATA6-; NANOG+ cells in green, double positive cells (DP) in yellow, and double negative cells (DN) in blue. n = 4, error bars indicate 95% confidence intervals. Scale bars: 20 μm in A, C.

Development • Supplementary information

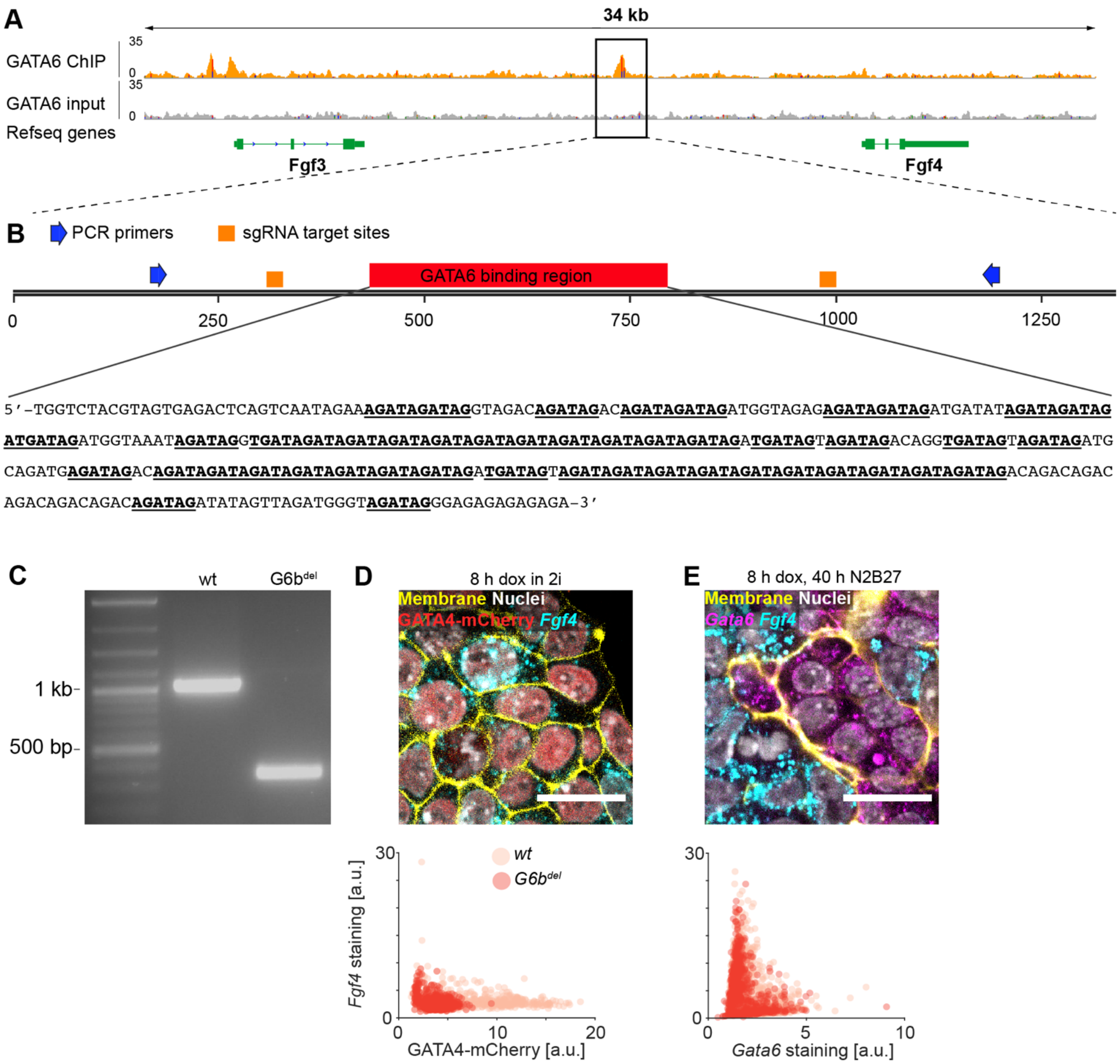

Fig. S4. Characterisation of a GATA6-binding site upstream of *Fgf4*. (A) ChIP-seq profile showing GATA6 binding upstream of *Fgf4*. The input control profile is included for comparison (gray). Raw data is from (Wamaitha et al., 2015). (B) Schematic overview (top) and nucleotide sequence (bottom) of the GATA6-binding region upstream of *Fgf4*. Sequences with the GATA6 consensus motif A/T-GATA-A/G are underlined and highlighted in bold. (C) PCR validation of successful deletion of the GATA6 binding site in inducible cells. Location sgRNAs used for deletion and PCR primers used for detection are indicated in B as orange boxes and blue arrows, respectively. $G6b^{del}$ indicates cell line with a deletion of the GATA6 binding site. (D) Top: GATA4-mCherry expression (red) and Fgf4 mRNA (cyan) in $G6b^{del}$ cells at the end of an 8 h doxycycline pulse. Nuclei stained with Hoechst33342 (white), cell membranes labeled with CellBrite (yellow). Bottom: Corresponding single cell analysis. Data from $G6b^{del}$ cells shown in dark red, same analysis for wild-type cells is reproduced from Fig. 2B and shown in light red for comparison. (E) Top: Gata6 (magenta) and Fgf4 mRNA (cyan) expression in $G6b^{del}$ cells after 40 h of culture in N2B27 following an 8 h doxycycline pulse. Nuclei stained with Hoechst33342 (white), cell membranes labeled with CellBrite (yellow). Bottom: Corresponding single cell analysis. Data from $G6b^{del}$ cells in dark red, same analysis for wild-type cells reproduced from Fig. 2C shown in light red for comparison. Scale bars: 20 µm in D, E.

Development • Supplementary information

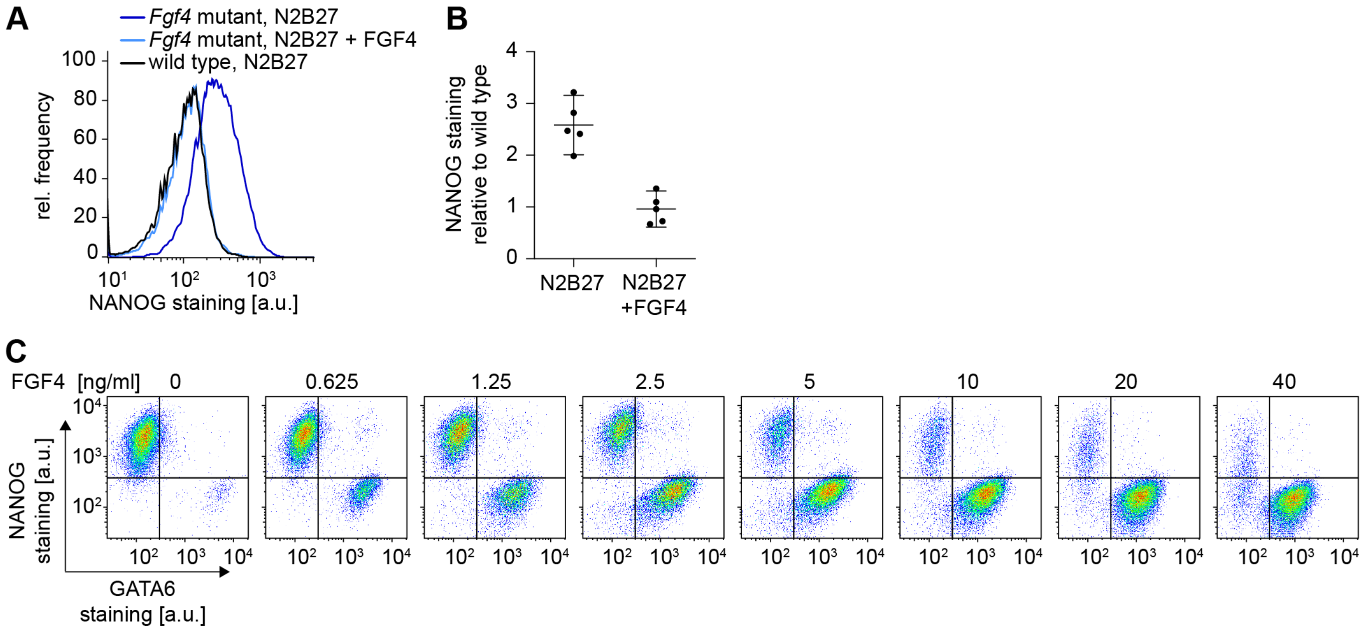

Fig. S5. Recombinant FGF4 rescues differentiation in *Fgf4*-mutant cells. (A) Flow cytometry histograms of NANOG expression in wild-type (black) or *Fgf4*-mutant (blue) cells after 40 h of culture in N2B27 with (light blue) or without recombinant FGF4 (dark blue, black). (B) Quantitative analysis of NANOG expression levels measured by flow cytometry of *Fgf4*-mutant cells cultured for 40 h in N2B27 with or without recombinant FGF4. Dots: Mean fluorescence intensities from ≥ 20.000 cells normalized to NANOG staining levels in the wild-type control in individual experiments. n = 4, error bars indicate 95% confidence intervals. **C** Flow cytometry profiles of *Fgf4*-mutant cells stained for NANOG and GATA6 after 8 h of doxycycline induction, followed by 40 h of differentiation in N2B27 medium supplemented with 1 µg/ml heparin and the indicated concentrations of recombinant FGF4. Lines indicate gates to assign cell types.

Development • Supplementary information

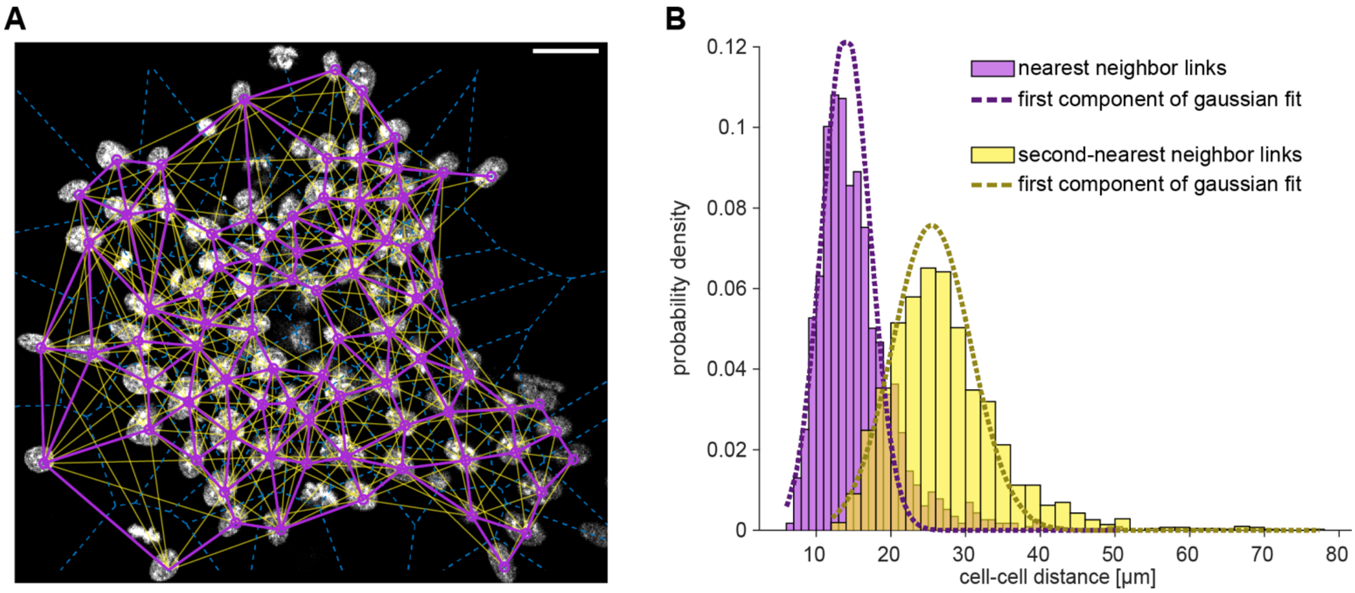

Fig. S6. Estimation of cell-cell distances by Delaunay triangulation. (A) Nuclear staining (white) of a colony 16 h after initiation of differentiation, overlaid with centres of mass of individual nuclei (purple circles), Voronoi edges (dashed blue lines), and links to nearest (purple) and second nearest neighbors (yellow, see Methods). Scale bar, 20 µm. (B) Histogram of distance distributions between nearest neighbors (purple) and second nearest neighbors (yellow), determined as in A. To separate true nearest and second-nearest neighbor distances from spurious longer-distance links, we applied two-component Gaussian mixture model fits to each of the distributions. Dashed lines indicate the first components of each fit. Distances determined by these first components were 14.0 ± 3.2 (mean ± s.d.) for nearest neighbors, and 25.5 ± 5.3 µm for second nearest neighbors. n = 8 independent fields of view.

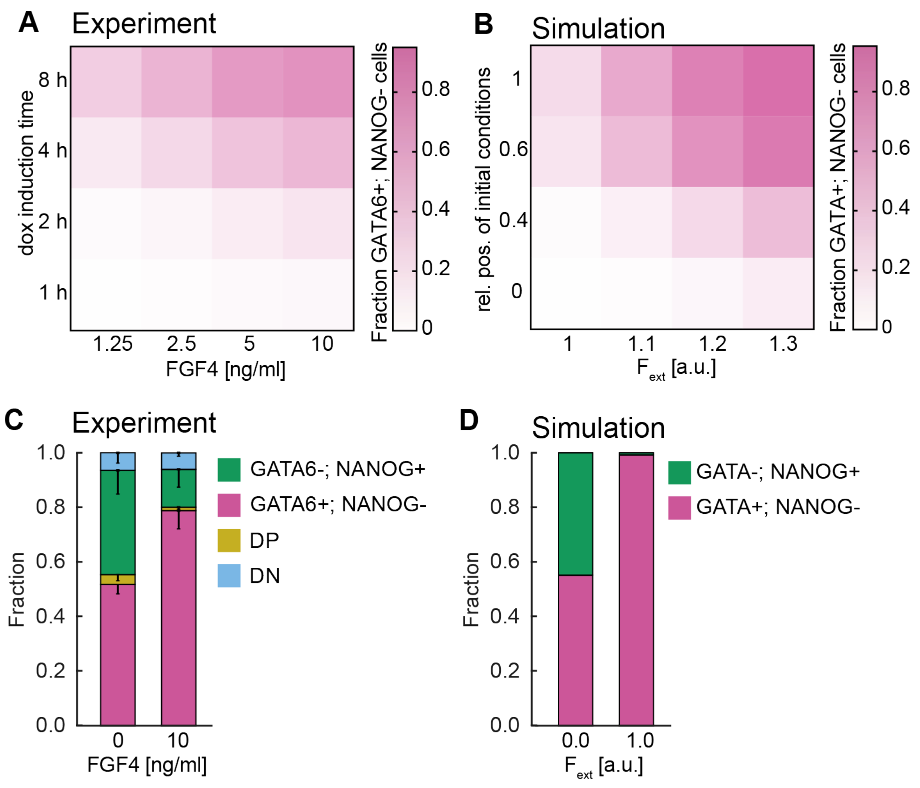

Fig. S7. FGF4-dependence of cell-type proportions in *Fgf4*-mutant and wild-type cells. (A) Proportion of GATA6+;NANOG- cells in *Fgf4*-mutant cultures after 40 h of differentiation in N2B27 as a function of prior doxycycline induction time (vertical axis) and FGF4 concentration during differentiation (horizontal axis). Each square corresponds to one combination of induction time and FGF4 dose, the proportion of GATA6+; NANOG- cells is color coded in hues of magenta. (B) Proportion of GATA+ cells in simulations of the single cell model for combinations of different initial conditions (vertical axis) and FGF signaling strengths (horizontal axis). Each square corresponds to one combination of initial conditions and FGF signaling strength, the proportion of GATA+ cells is color coded in hues of magenta. (C) Cell-type proportions in wild-type GATA4-mCherry inducible cells after an 8 h doxycycline pulse followed by differentiation in N2B27 alone (left) or in N2B27 supplemented with 10 ng/ml FGF4 (right), measured by flow cytometry. Fraction of GATA6+, NANOG- cells in magenta, GATA6-, NANOG+ cells in green, double positive cells (DP) in yellow, and double negative cells (DN) in blue. n = 4, error bars indicate 95% confidence intervals. (D) Cell-type proportions in simulations of the coupled model without (left), or with addition of an exogenous FGF signal (right). Initial conditions were chosen such that NANOG and GATA had similar expression levels. Data in C, D for conditions without exogenous FGF signal is reproduced from Fig. 4C and 4G for comparison.

Development • Supplementary information

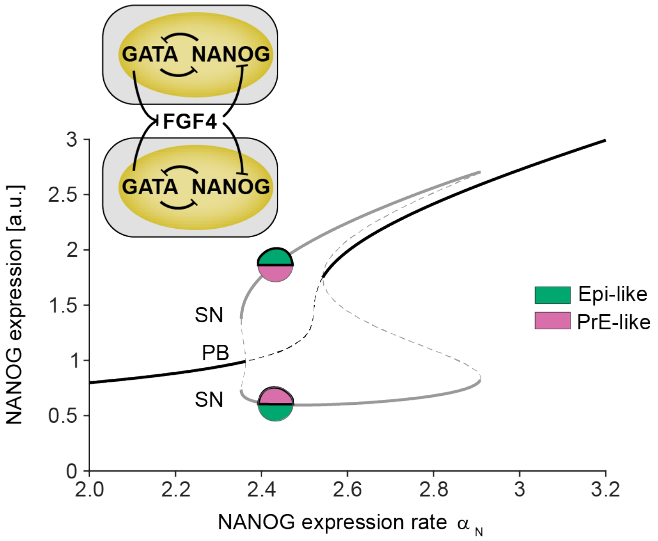

Fig. S8. Bifurcation analysis of a 2-cell coupled model. Bifurcation diagram showing the principle of emergence of a symmetry-broken inhomogeneous steady state (IHSS) for a two-cell coupled system (inset) in dependence of the production rate constant of NANOG (αN), using NANOG expression as a representative variable. The IHSS (gray lines) is generated via a pitchfork (PB) and stabilized via saddle-node (SN) bifurcations. The two branches represent the heterogeneous attractors: (GATA6-; NANOG+) for cell 1 and (GATA6+; NANOG-) for cell 2, or (GATA6+; NANOG-) for cell 1 and (GATA6-; NANOG+) for cell 2 (representative circles). Solid/dashed lines indicate stable/unstable solutions. Black: homogeneous steady state; gray: IHSS solution.

Development • Supplementary information

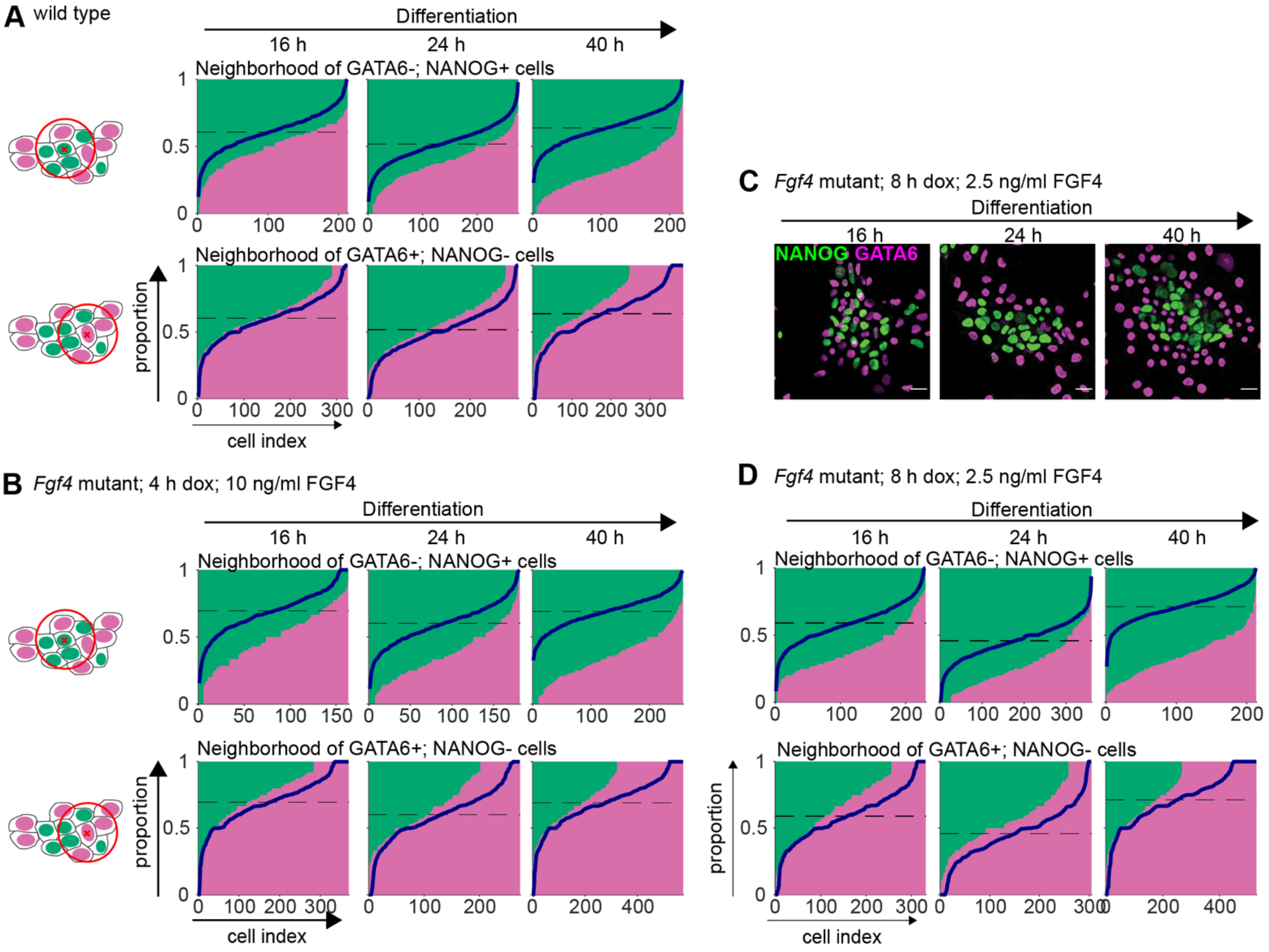

Fig. S9. Cell-type composition of local cell neighborhoods. (A) Cell-type proportions in a local neighborhood of 31.7 μm diameter in wild-type cells stimulated for 8 h with doxycycline and differentiated for the indicated times. Each column in a panel corresponds to one cell, and the sizes of its associated green and magenta bars correspond to the fraction of GATA6-; NANOG+ (green) and GATA6+; NANOG- (magenta) cells in its local neighborhood. Blue lines indicate the corresponding random distribution, calculated by assigning the global distribution of cell types of the time point randomly to experimentally determined cell positions. Clustering of cell types results in the deviation of the true proportions from this random distribution. The local neighborhood of GATA6-; NANOG+ cells (top row) indicates clustering of this cell type already at 16 h, which becomes more pronounced over time. Strong clustering of GATA6+; NANOG- cells (lower row) can only be observed at 40 h. (B) Same analysis as in A, but for *Fgf4*-mutant cells induced for 4 h and differentiated in N2B27 supplemented with 10 ng/ml FGF4 for the indicated periods of time. Deviation from random arrangement (blue lines) indicates clustering of GATA6-; NANOG+ cells at 16 h, which becomes more pronounced over time. Some clustering of GATA6+; NANOG- cells (lower row) can already be detected at 16 h, and becomes more pronounced until 40 h. (C) Immunostaining for NANOG (green) and GATA6 (magenta) in *Fgf4*-mutant cells induced with doxycycline for 8 h and differentiated in N2B27 supplemented with 2.5 ng/ml FGF4 for the indicated periods of time. (D) Same analysis as in A, B, for *Fgf4*-mutant cells differentiated as described in **C**. Deviations from random arrangement (blue lines) are similar to those seen in B, indicating similar clustering dynamics.

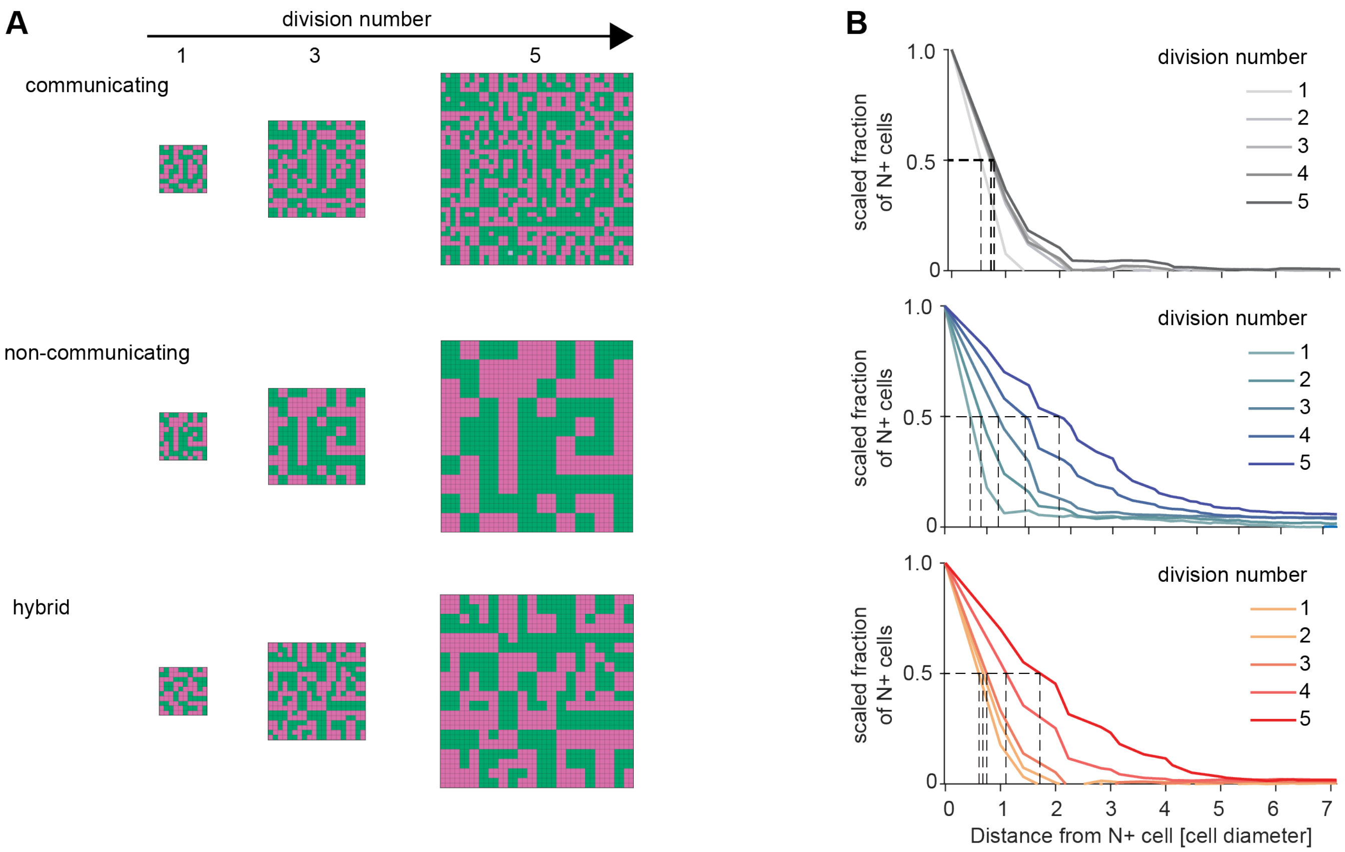

Fig. S10. Spatial patterns of cell types in model simulations. (A) Spatial configurations of cell types in single numerical realizations of the model with cell-cell communication (top row), without cell-cell communication (middle row) and of a hybrid model (bottom row), in which communication is switched off after the third division. First column shows cell-type pattern at the end of the first cell cycle (grid size 10 x 10), second column shows pattern at the end of the third cell cycle (grid size 20 x 20), and third column shows patterns at the end of the fifth cell cycle (grid size 40 x 40). (B) Corresponding estimation of NANOG cluster radius for single numerical realizations of the model variants shown in A. Graphs depict the scaled fraction of N+ cells within a specific radius around seed cells. Dashed lines indicate determination of cluster radius, the distance around NANOG+ cells at which the scaled fraction is equal to 0.5. Cluster radii were obtained after each cell cycle of the lineage tree simulation.

Development • Supplementary information

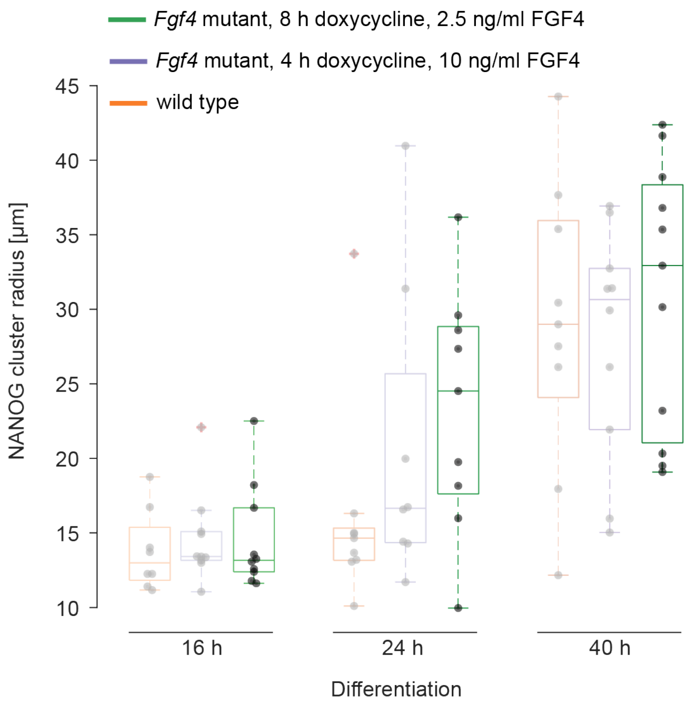

Fig. S11. Cluster radius dynamics in *Fgf4*-mutant cells are independent from stimulation and differentiation regime. Dark: Cluster radii of N+ cells in *Fgf4*-mutant cultures induced with doxycycline for 8 h, followed by differentiation in N2B27 medium supplemented with 2.5 ng/ml FGF4 for the indicated times. Dots indicate values from n ≥ 9 individual fields of view from the same experiment, box plots show median, interquartile ranges and outliers (red cross). Shaded data points are reproduced from Fig. 5C for comparison, and show cluster radii for wild-type cells differentiated in N2B27 only (orange), and for *Fgf4*-mutant cells differentiated in N2B27 supplemented with 10 ng/ml FGF4 following 4 h of induction with doxycycline (blue).

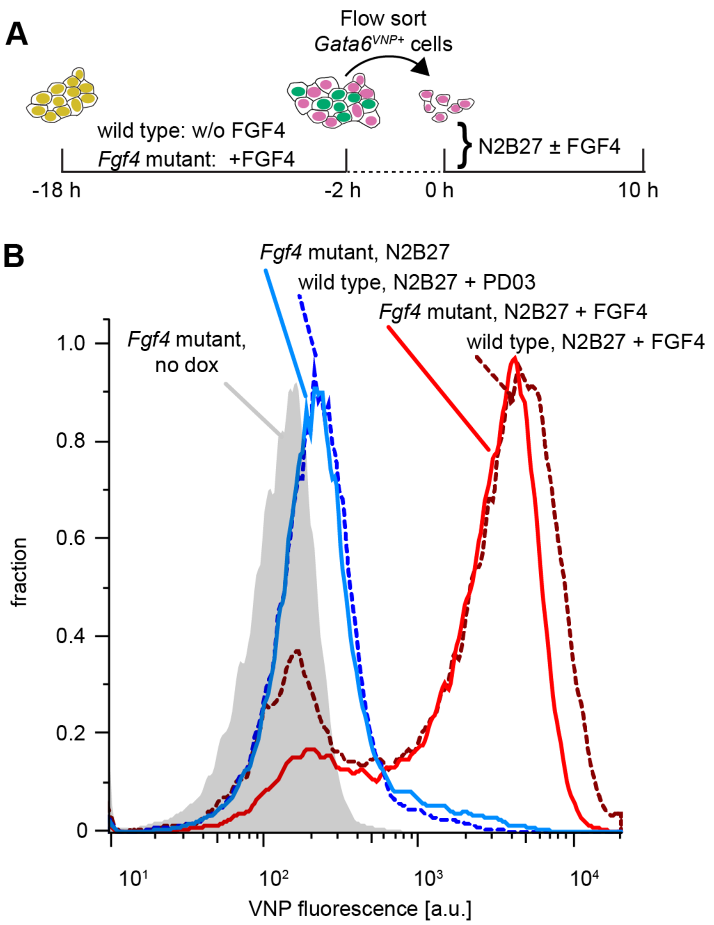

Fig. S12. Gata6^{VNP} reporter expression in sorted *Fgf4*-mutant cells. (A) Schematic representation of the cell-sorting experimental protocol for *Fgf4*-mutant cells. Following a doxycycline pulse, *Fgf4*-mutant cells were initially differentiated in medium supplemented with FGF4, while wild-type cells were differentiated without recombinant FGF4. After flow sorting of VNP positive cells at 16 h, wild-type and *Fgf4*-mutant cells were cultured in N2B27 medium with or without FGF4, followed by analysis of VNP expression. (B) Flow cytometry histograms of VNP expression in wild-type (dashed lines) and *Fgf4*-mutant (solid lines) inducible Gata6^{VNP} cells, 10 h after sorting of VNP-positive cells and culture in the indicated media.

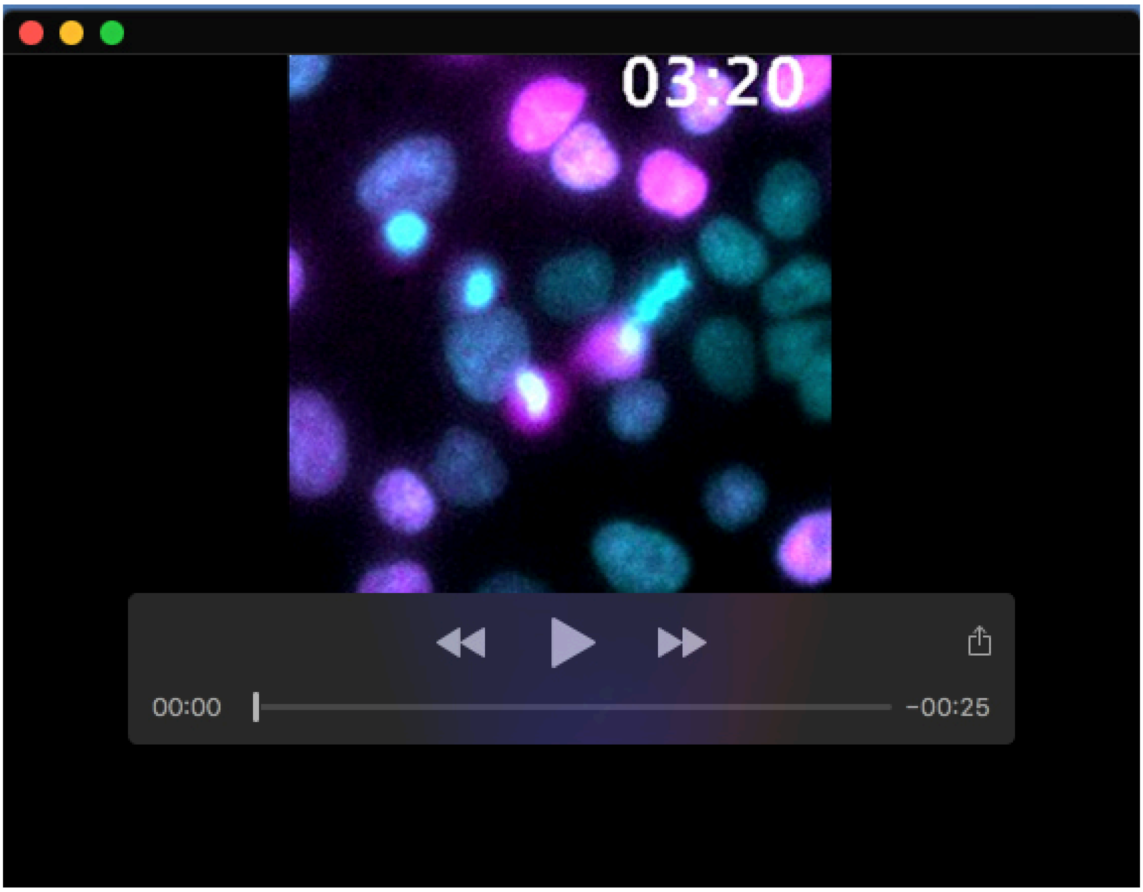

Movie 1. Dynamics of VNP expression in unperturbed colonies
Time-lapse imaging of a colony of Gata6^{VNP} reporter cells starting 16 h after the end of a doxycycline pulse. Medium has been switched to N2B27 at the beginning of the recording. Frame rate is 10 min.

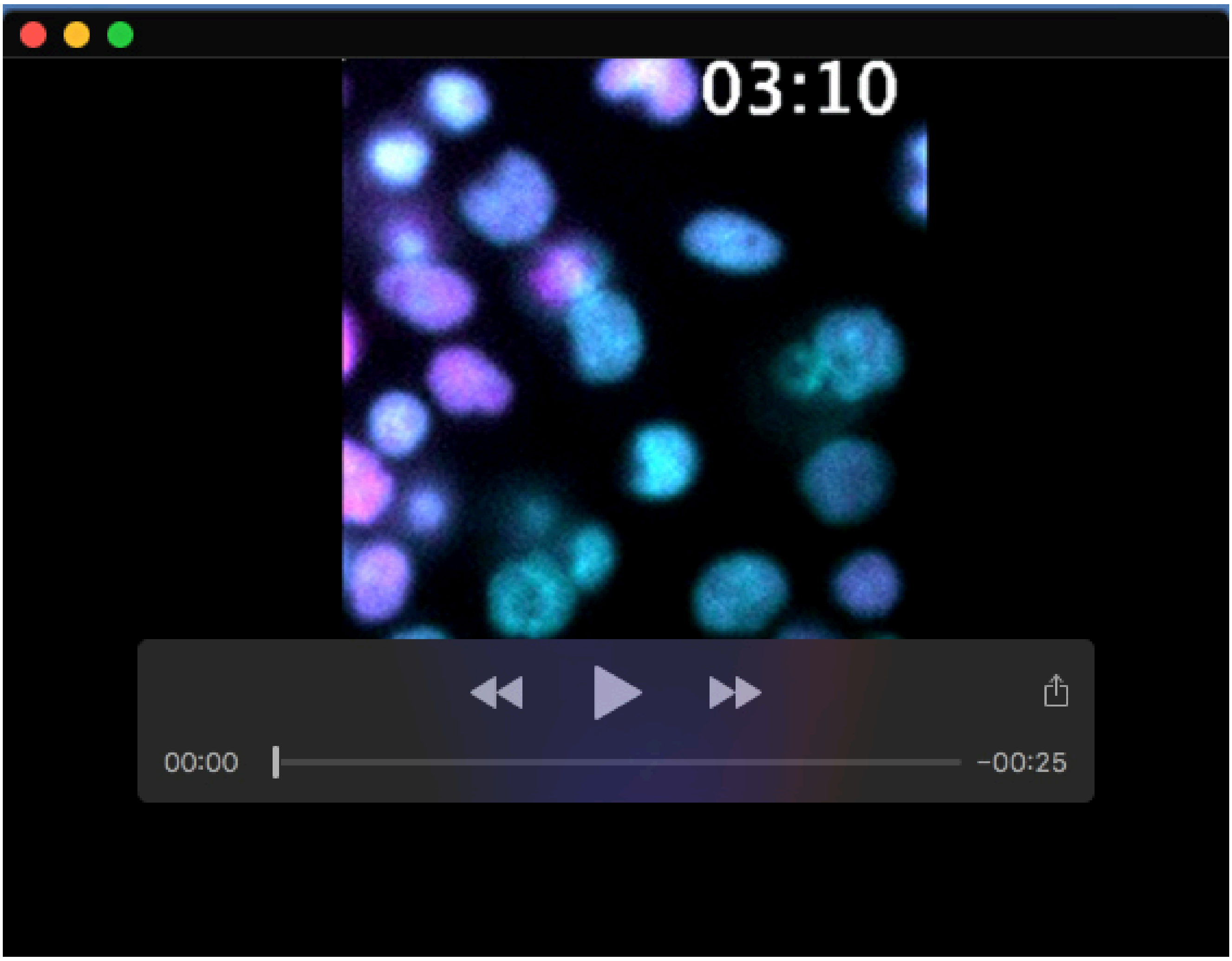

Movie 2. Dynamics of VNP expression sorted Gata6^{VNP}-positive cells cultured in N2B27.
Time-lapse imaging of Gata6^{VNP} reporter cells flow sorted for VNP expression 16 h after the end of a doxycycline pulse and cultured in defined N2B27 medium alone. Frame rate is 10 min.

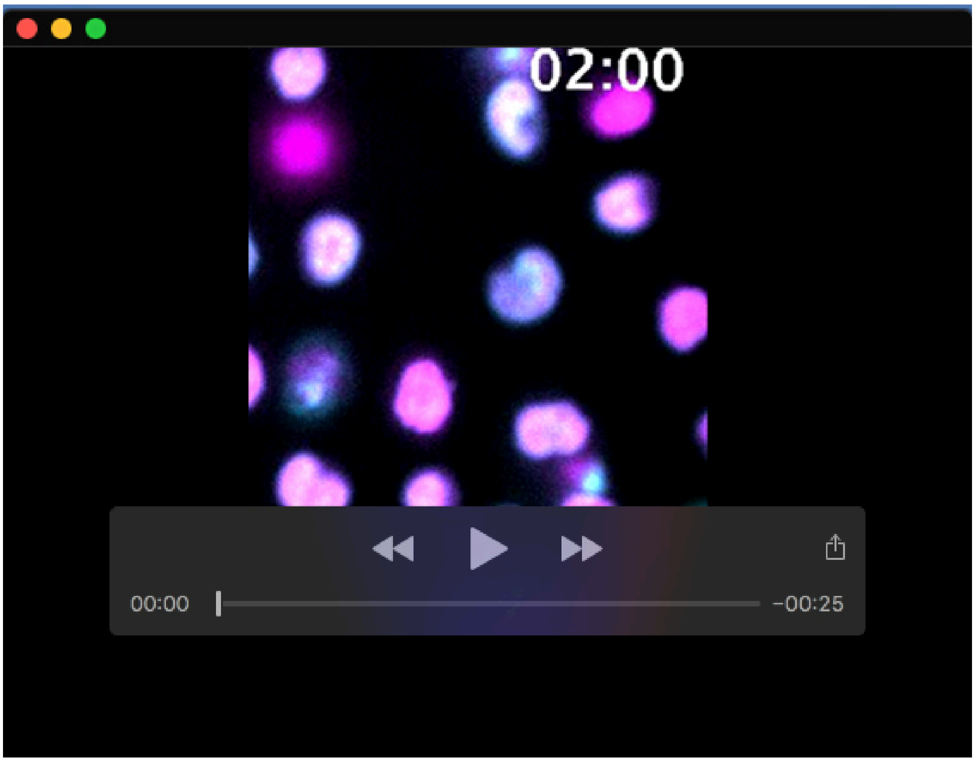

Movie 3. Dynamics of VNP expression sorted Gata6^{VNP}-positive cells cultured in N2B27 supplemented with FGF4. Time-lapse imaging of Gata6^{VNP} reporter cells flow sorted for VNP expression 16 h after the end of a doxycycline pulse and cultured in defined N2B27 medium supplemented with 10 ng/ml FGF4. Frame rate is 10 min.

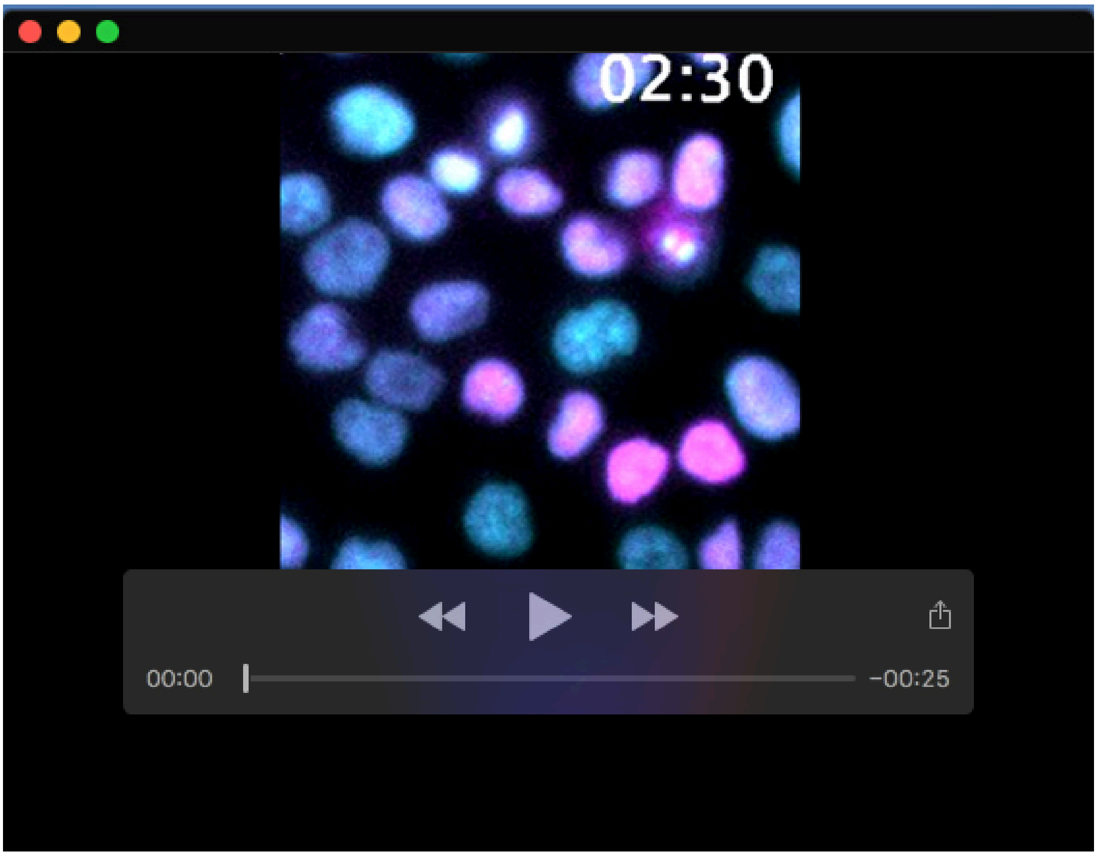

Movie 4. Dynamics of VNP expression sorted Gata6^{VNP}-positive cells cultured in N2B27 supplemented with PD03. Time-lapse imaging of Gata6^{VNP} reporter cells flow sorted for VNP expression 16 h after the end of a doxycycline pulse and cultured in defined N2B27 medium supplemented with 1 μM of the MEK inhibitor PD03. Frame rate is 10 min.
