# Supplementary material for: Cell-cell communication through FGF4 generates and maintains robust proportions of differentiated cell types in embryonic stem cells
Source: Development. 2021 Nov 5;148(21):dev199926. doi: 10.1242/dev.199926 (PMC8602943; doi:10.1242/dev.199926)
Supplement: Reviewer comments [file develop-148-199926_review_history.pdf]

Cell-cell communication through FGF4 generates and maintains robust proportions of differentiated cell types in embryonic stem cells

Dhruv Raina, Azra Bahadori, Angel Stanoev, Michelle Protzek, Aneta Koseska and Christian Schröter
DOI: 10.1242/dev.199926

Editor: James Briscoe

Review timeline
Original submission: 22 June 2021
Editorial decision: 24 June 2021
First revision received: 13 September 2021
Accepted: 4 October 2021

Original submission

First decision letter

MS ID#: DEVELOP/2021/199926

MS TITLE: Cell-cell communication through FGF4 generates and maintains robust proportions of differentiated cell types in embryonic stem cells

AUTHORS: Dhruv Raina, Azra Bahadori, Angel Stanoev, Michelle Protzek, Aneta Koseska, and Christian Schroter

Thank you for submitting your manuscript via Review Commons. You can access the files online: please go to BenchPress and click on the 'Manuscripts with Decisions' queue in the Author Area.

The referees express considerable interest in your work, but they do have some significant criticisms and recommend a substantial revision of your manuscript before we can consider publication. Overall, the referees's criticisms are constructive and your proposals to address the individual points are reasonable. I note that you propose to investigate the functional significance of the GATA binding site upstream of Fgf4. While I think these experiments are interesting and will add a further dimension to your study I don't think they are strictly necessary to support the conclusions of the current study. I will leave it to your discretion whether to include these. Reviewer 2 suggests assaying GATA6 to infer the range of FGF4 signaling (Point 9), I agree that this would strengthen your conclusion and I would encourage you to include this analysis. Both Reviewer 1 and 3 raise the question of the regulatory interaction between Nanog and FGF4, your reasoning for excluding this repression in the model should be discussed.

If you are able to revise the manuscript along the lines suggested, which may involve further experiments, I will be happy receive a revised version of the manuscript. Your revised paper will be re-reviewed by one or more of the original referees, and acceptance of your manuscript will depend on your addressing satisfactorily the reviewers' major concerns. Please also note that Development will normally permit only one round of major revision.

We are aware that you may be experiencing disruption to the normal running of your lab that make experimental revisions challenging. If it would be helpful, we encourage you to contact us to discuss your revision in greater detail. Please send us a point-by-point response indicating where

© 2021. Published by The Company of Biologists under the terms of the Creative Commons Attribution License (https://creativecommons.org/licenses/by/4.0/).

you are able to address concerns raised (either experimentally or by changes to the text) and where you will not be able to do so within the normal timeframe of a revision. We will then provide further guidance. Please also note that we are happy to extend revision timeframes as necessary.

Please attend to all of the reviewers' comments and ensure that you clearly highlight all changes made in the revised manuscript. Please avoid using 'Tracked changes' in Word files as these are lost in PDF conversion. I should be grateful if you would also provide a point-by-point response detailing how you have dealt with the points raised by the reviewers in the 'Response to Reviewers' box. If you do not agree with any of their criticisms or suggestions please explain clearly why this is so.

First revision

Author response to reviewers' comments

We would like to thank all three Reviewers for their constructive criticism which has helped us significantly in improving our manuscript. Below we describe point-by-point how we have addressed each of the Reviewer's comments through additional figures or through re-writing of the text. In the revised version, we have split the original Fig. 3, such that the figure numbering has changed relative to the original manuscript. The numbering of some of the supplementary figures has also changed. In this response, we refer to all figures using the numbering in the revised manuscript.

Reviewer #1 (Evidence, reproducibility and clarity (Required)):

In this manuscript Raina et al. use an in vitro model of PE specification based on the transient overexpression of GATA4 in ESCs to show that the acquisition of primitive endoderm (PE) identity is governed at the population levels by cell-cell interactions mediated by FGF signaling. The authors further argue that the specification of a defined proportion of "PE" and "Epiblast" cells in a differentiating population of ESC is an emergent property of a system where paracrine signaling shifts the balance between two alternative stable states. Overall, the work does not reach radically new conclusions: broadly similar models are outlined in several other publications, including from the authors. Yet this study makes use of elegant genetic models and is particularly well executed. In addition, it includes a very accurate characterisation of the spatial range of FGF signaling activity that is original and adds on the existing knowledge. Moreover, the authors show novel evidence suggesting that GATA factors inhibits Fgf4 transcription and the activity of the FGF signaling pathway in ESCs.

Two major points deserve further clarification:

1. In this manuscript the authors claim that the proportions of cells acquiring PE fate is, at least in the experimental setup adopted, largely independent from the levels of GATA4 induction, and therefore of the initial state of the gene regulatory network regulating this cell fate transition. However, the authors should discuss how the current findings relate to their previous results, showing that the duration/levels of Gata4 induction, in a similar experimental setting, play an important role in determining the final proportion of cells cell acquiring "PE" fate. Absolute expression levels may be crucial for this distinction, but the authors seem to exclude this possibility (see figure S3).

The different roles of GATA4-mCherry induction levels for determining the final proportion of cells acquiring a PrE-like fate reported in our previous (Schröter et al., 2015) and the current work is because of important differences in the experimental settings between the two studies. In Schröter et al., 2015, we assayed PrE-like differentiation in medium supplemented with serum and LIF, which provides exogenous signals that promote PrE-like differentiation. Under these conditions, we revealed the function of the cell-autonomous circuit, in which GATA4-mCherry levels do control the probability of PrE- like differentiation. In the current work, we use a defined medium without exogenous growth factors to reveal the population-level behavior of communicating cells. At the population-level, we find emergent behavior in which cell type

© 2021. Published by The Company of Biologists under the terms of the Creative Commons Attribution License (https://creativecommons.org/licenses/by/4.0/).

proportions are independent from GATA4-mCherry induction levels. We highlight and discuss these critical differences between our previous and the current work and the novel insight that they bring in several places throughout the revised manuscript, e.g. lines 84 - 87, 133, and 421 - 424.

2. Most importantly, the authors incorporate in their model the notion that GATA6 inhibits FGF signaling. It would be interesting to understand how such inhibition is mechanistically mediated. For instance GATA6 has been shown to bind in proximity of the Fgfr2 gene (Wamaitha et al., Genes and Dev., 2015). Alternatively, the authors show a direct effect on Fgf4 expression. The short time window of the reported repressive transcriptional effects (8h, Fig 2 middle), might suggest a direct regulation. The authors should test this possibility, and discuss what alternative modes of regulation could be envisaged (for instance, indirect effects mediated by Nanog). This is a key result that deserves a more detailed mechanistic characterisation.

Prompted by the reviewer's comment, we have added new experiments and analyses to the revised manuscript to pinpoint how the inhibition of FGF signaling by GATA factors is mechanistically mediated. Specifically, we show that Fgf4 mRNA levels decrease rapidly upon induction of GATA4-mCherry, much earlier than NANOG protein levels (new Fig. 2B). In contrast, Fgf4 remains expressed upon culture of cells for 40 h in N2B27 without doxycycline induction, even though NANOG is almost completely downregulated under these conditions (new Fig. 2C, D). We also identify a GATA6 binding site upstream of the Fgf4 gene in a published ChIP-seq dataset (Fig. S4A, B). Even though Fgf4 is still repressed upon GATA induction in a cell line where this putative GATA6 binding site has been deleted (Fig. S4 C, D), the new data together make a strong point for direct repression of Fgf4 by GATA factors, and thereby establish a new regulatory link in the signaling network underlying the differentiation of Epi- and PrE-like cells. The new data is discussed in lines 170 - 192 and 430 - 435 of the revised manuscript.

Minor points:

Fig S1: The authors should show quantifications of Nanog and GATA6 levels before the beginning of the differentiation protocol.

We have added this data in two new panels A and B in Fig. S1. Also see our response to the next point below.

Line 106: The authors write "the initially large proportion of GATA6+; NANOG+ double positive cells". It appears that at 16h of differentiation ESCs have already partitioned between Gata6 or Nanog expressing cells. The authors should rephrase the sentence to reflect what seems to be an almost total absence of truly double positive cells. Possibly, an analysis conducted at earlier time points could clarify these dynamics.

To address this and the previous point, we have performed immunostaining with GATA6-specific antibodies of cells cultured in 2i + LIF medium, after 8 h of doxycycline induction, and at 8h and 16 h after the beginning of differentiation. These data are shown in Fig. S1A,B. While endogenous GATA6 is not expressed in 2i + LIF and immediately after 8 h of doxycycline induction, weak GATA6 expression can be detected 8 h later. At this time point, NANOG and GATA6 expression is most heterogeneous in the population, and some cells truly co-express the two proteins. As rightly pointed out by the reviewer, this co-expression is largely resolved at 16 h after the start of differentiation. We have updated the text to discuss these new results, and rephrased our description of the expression pattern at 16 h accordingly (lines 106 - 110).

Line 124: The authors write "... concentration dependent downregulation of NANOG expression". The effects may rather depend on the time of doxycycline stimulation.

We have rephrased this sentence and now state that "...NANOG expression levels decreased with longer doxycycline induction time." (lines 124, 125). We note that in the independent clonal lines, where induction time is constant, NANOG repression is negatively correlated with GATA4-mCherry levels (Fig. S3A). Thus, these cell lines do demonstrate purely concentration-dependent effects of GATA4-mCherry expression, and we now motivate the section describing Fig. S3 along those lines (line 144).

© 2021. Published by The Company of Biologists under the terms of the Creative Commons Attribution License (https://creativecommons.org/licenses/by/4.0/).

Line 192: The authors write "...and confined to cells with low GATA4-mCherry expression levels". It would be helpful to have an indication of the cell boundaries, possibly showing localisation of a membrane bound protein.

In the new Fig. 2B, we have now labelled cell membranes with a CellBrite dye to outline cell boundaries. Use the membrane labelling, we have performed single-cell quantifications of Fgf4 mRNA and GATA4- mCherry expression which further support our point that Fgf4 mRNA expression is negatively correlated with GATA4-mCherry expression in individual cells.

It would be interesting for the authors to discuss how the spatial range of FGF activity measured in culture could affect PE specification in the embryo.

In lines 439 - 446 of the revised manuscript, we now discuss how the short spatial range of FGF activity in culture could explain why the specification of Epi and PrE cells in the ICM is initially spatially random, followed by a sorting step.

Reviewer #1 (Significance (Required)):

See above.

Reviewer #2 (Evidence, reproducibility and clarity (Required)):

In their manuscript entitled "Cell-cell communication through FGF4 generates and maintains robust proportions of differentiated cell types in embryonic stem cells" Raina et al study the effect of Fgf- signalling based local cell-cell communication for the establishment of PrE-like and Epi-like cells. The authors use an elegant, albeit artificial, system to analyse the effect of Fgf signalling on establishing 'normal' lineage proportions after transient induction of Gata4 expression. The main conclusions of the manuscript are: i) Gata6 positive cells emerge through short range Fgf4 based cell-cell cummunication.
ii) Fgf4 signalling can compensate a wide range of initial levels of Gata6 expression and produce properly portioned cell identities. The authors also state that this mechanism could operate in a range of developing tissues.

Major points:

1. Fgf4 KOS ESCs are deficient in initiating epiblast lineage differentiation (Kunath 2007). Therefore, the effect studied by the authors might be multifactorial and the general inability of Fgf4 deficient cells to enter differentiation might contribute to the observed differentiation defects and defects of cell fate proportioning. Specifically, it could be expected that Nanog regulation is affected in Fgf4 mutants, although, to my knowledge, the specific phenotype of Fgf4 depletion has not been evaluated in Gata4 induced cell programming towards PrE. What steps have the authors taken to exclude an impact of general cell fate change defects in Fgf4 KO ESCs.

The reviewer rightly points out that *Fgf4* mutant cells are not only deficient in PrE-like differentiation but also have previously been shown to be defective in epiblast-like differentiation. In the revised manuscript, we acknowledge this general differentiation deficiency and use flow cytometry for NANOG staining to show that it is recapitulated in our mutant line (new panel A in Fig. S5 and lines 195 - 198). Here we also show that addition of recombinant FGF4 fully rescues NANOG regulation. This is important, as it indicates that by addition of FGF4, we can generate a situation where we rescue the general cell fate change effects in *Fgf4* mutant cells, and thus separate them from the deficiency of this cell line to communicate within a cell population. This is an important conceptual point, which we further emphasize in lines 260 - 263 of the revised manuscript. The regulation of NANOG expression by FGF4 mentioned by the Reviewer is represented in our model.

2. Increasing the time of Gata4 expression results in increasing levels of Gata4 levels (Fig 1C). This is shown at the overall mean fluorescence level. However, it is important to also quantify how many cells do actually show some increase in Gata4 levels. Fig1D suggests that the number of Gata4 expressing cells is quite similar between 4h and 8h induction, but this needs to be quantified.

© 2021. Published by The Company of Biologists under the terms of the Creative Commons Attribution License (https://creativecommons.org/licenses/by/4.0/).

An explanation for the apparent dosage independence of Gata4 could then be simple threshold effects, such that there is no additional effect of increased Gata4 levels in WT cells without any further requirement of feedback regulation after a certain threshold level of Gata4 is reached. Have the authors considered such a simple model?

We have quantified GATA4-mCherry expression levels in single cells after different induction times in Fig. S2A. This analysis confirms the Reviewer's visual impression of Fig. 1D - the number of GATA4- mCherry expressing cells is similar for different induction times and clonal lines, such that the increase in overall mean fluorescence levels is mainly due to an increase in GATA4-mCherry expression levels in single cells. In the revised manuscript, we explicitly point the reader to this important piece of data (lines 125, 126 for the titration of GATA4-mCherry levels by varying doxycycline induction time, and line 147 for the quantification of GATA4-mCherry levels in the independent clonal lines). The Reviewer's suggestion of a simple threshold effect is further ruled out through our experiments where we add recombinant FGF4 to wild type cells during differentiation (Fig. S2C and Fig. S3D). This leads to a systematic shift of cell type proportions with higher GATA4-mCherry induction levels, and thus shows that GATA4-mCherry levels are functionally relevant as soon as we override the endogenous communication mechanism. This motivation of the FGF4-addition experiment is stated explicitly in lines 135 - 138 of the revised manuscript.

An important point is that in the current setup distinguishing between dosage effects and effects of extended presence of Gata4 cannot be distinguished. Wouldn't titrating the amount of doxycycline used for induction be a more direct way to achieve different initial levels of Gata4 expression?

We agree with the Reviewer that titrating GATA4-mCherry levels by varying doxycycline induction times does not allow distinguishing between dosage effects and effects of extended presence of Gata4. The results shown in Fig. S3, where we use clonal lines with independent integrations, were intended to specifically address this point. In these lines, the duration of doxycycline induction and hence time of GATA4-mCherry exposure is held constant, such that here, the only difference between the conditions is GATA4-mCherry dosage (Fig. S3). The Reviewer's comment indicates to us that the motivation for the experiments in Fig. S3 had not been made sufficiently clear in the original manuscript. We have therefore re-written the introductory sentence to this Figure (line 144), and hope that this addresses the Reviewer's point.
Titrating doxycycline does unfortunately not allow titrating transgene induction levels in a meaningful way, as sub-saturating doses of doxycycline lead to an increased heterogeneity in transgene expression with many non-expressing cells, rather than to reduced expression levels across all cells. PMID: 17048983 offers a possible explanation of this observation.

Another point the authors should appropriately discuss and consider is that a lack of effect of different doses/durations of Gata4 expression could be due to the fact that by the time Gata6 is induced, the levels of Gata4 in cells previously treated for different periods of time are no longer detectably different. Such a regulation would equally result in indistinguishable cell fate proportioning. Can the authors exclude such a regulation? This is an important point at the heart of the authors conclusion.

The Reviewer suggests that by separating the initiation of GATA6 expression from the GATA4-mCherry pulse in time, the decision to initiate PrE-like differentiation could be independent from GATA4-mCherry concentration, thus explaining the robust cell type proportions. Our new analysis of the expression dynamics of GATA4-mCherry and endogenous GATA6 after a doxycycline pulse indicates that both proteins are simultaneously expressed 8 h after an 8 h doxycycline pulse (new panels A and B in Fig. S1). Thus, GATA4-mCherry and endogenous GATA6 expression are not strictly separated in time. More importantly, the experiments where we add recombinant FGF4 to wild type cells during differentiation (Fig. S2C and Fig. S3D) rule out the possibility that GATA4-mCherry levels are not relevant for cell type proportions. Exogenous FGF4 leads to a systematic shift of cell type proportions with higher GATA4- mCherry induction levels, demonstrating that GATA4-mCherry levels are functionally relevant as soon as we override the endogenous communication mechanism. As already mentioned in our response to point 2. above, this line of reasoning is now explicitly stated in lines 135 - 138 as well as lines 142 -143 of the revised manuscript.

© 2021. Published by The Company of Biologists under the terms of the Creative Commons Attribution License (https://creativecommons.org/licenses/by/4.0/).

3. The authors make some general statements on cell differentiation (e.g. l205). They also claim that the Fgf4-based mechanism of lineage proportioning could act in a range of tissues during development. However, the use of the term differentiation for the induction of PrE-identity (or Gata-factor expression to be exact, see comment below) after Gata4 overexpression is problematic. The system chosen by the authors is entirely artificial. ES cells normally do not differentiate into extraembryonic cell types. It needs to be made clear in the manuscript that they do not study a differentiation process that normally occurs in the embryo or in differentiating ESC cultures. The system the authors are using would, in my opinion, rather qualify as cell programming or transdifferentiation than as differentiation. I suggest presenting the system using clearer unambiguous language and to try to avoid any generalisations based on an artificial transgene-overexpression based system. The results have to be presented with this limitation in mind.

In lines 415 - 417 of the revised manuscript, we now acknowledge explicitly that PrE-like cells do not spontaneously emerge in ESC cultures, as correctly pointed out by the Reviewer. We still think however that the term “differentiation” correctly captures the acquisition of discrete cell identities that we observe upon transient GATA expression, and therefore decided to keep using this term throughout our manuscript. The value of the experimental system to delineate general mechanisms of cell type proportioning is also supported by Reviewer #3 in the referee’s cross commenting section.

4. It is unclear how 'PrE-like' (as stated e.g. in the abstract) the cells really are after a short pulse of Gata4 expression. No proper characterisation has been performed but needs to be included, if the authors want to term these cells PrE-like.

To address this point, we have included new immunostainings for the PrE markers SOX17 and Laminin in the revised manuscript (new panels E and F and Fig. S1). SOX17 expression, like GATA6 expression, is mutually exclusive with NANOG expression, and GATA6-positive cells show high levels of Laminin. We also note that a short pulse of GATA4 expression was used to induce PrE-like differentiation in a recent study by Amadei et al. (PMID: 33378662). In this study it was shown that the resulting cells recapitulated functional properties of the embryonic primitive endoderm, and differentiated into more specialized cell types such as the anterior visceral endoderm. We therefore think it is justified to refer to the GATA6-positive cells in our study as “PrE-like”.

5. How is the statement in l112 that "The clear separation between the two populations suggests that the increase in the proportion of double negative cells at the expense of GATA6+; NANOG- PrE-like cells beyond 40 h is mostly fueled by the downregulation of NANOG expression in the GATA6-negative cell population, combined with a slower proliferation of the GATA6-positive population, rather than by the reversion of PrE-like into double negative cells." supported by the data?

We realised from the comments of all three Reviewers that this section was confusing and potentially misleading in the original version of the manuscript. The main goal for analysing cell type proportions after increasing differentiation times was to test if and when these proportions would stabilize. To address this question more directly, we have now applied a different gating strategy to the flow cytometry data shown in Fig S1C. Specifically, we used a Gaussian mixture model to determine how many cells could be unambiguously assigned to the two main clusters in the NANOG/GATA6 expression space. This analysis reveals that the two clusters are fully segregated 40 h after the beginning of differentiation. The low number of cells in between the clusters indicates that cells rarely transition between the clusters. Still, we find that the proportion of PrE-like cells decreases from 40 h onwards. We interpret this as the consequence of a slower proliferation of this cell population. We also notice that NANOG expression in the GATA6-negative population is downregulated over time. This parallels NANOG downregulation in the embryonic epiblast and thus reflects the Epi-like character of this cell population (see also our response to Reviewer #3 below). These new analyses and interpretations are described in lines 109 - 114 of the revised manuscript.

6. Would the data and modelling performed by the authors be in line with a model in which the decision to express Gata6 is a stochastic choice (with a certain probability based on the levels of Gata4 induction) that is then stabilized and reinforced by Fgf signalling rather than Fgf signalling having an instructive role?

© 2021. Published by The Company of Biologists under the terms of the Creative Commons Attribution License (https://creativecommons.org/licenses/by/4.0/).

The simulations shown for the Fgf4 mutant case in Fig. 4D-G, right column, are based on a model in which the decision to express Gata is a stochastic choice with a probability based on the initial levels of GATA expression, and reinforced by FGF signaling. Thus, our data from the Fgf4 mutant, but not the wild type, are perfectly in line with such a model.
We realize from the Reviewer's question that we have not made sufficiently clear the critical differences between the models for the mutant and the wild type case. We have therefore added a more direct comparison of the two conditions in lines 260 - 263 of the manuscript. Furthermore, we explicitly state that the model for the rescued Fgf4 mutant effectively captures the single cell behavior where cell differentiation is exclusively governed by the dynamics of the mutually repressive NANOG-GATA circuit (lines 281 - 284 of the revised manuscript).

7. The statement in line 187 "This indicates that GATA4-mCherry expression negatively regulates FGF4 signaling during cell type specification." is not supported by the data. The authors show only a correlation and actually correctly say so in line 195.

We have re-written this statement (lines 170, 171 of the revised manuscript). We also provide additional data which suggest that the correlation between higher GATA4-mCherry levels and lowered FGF signaling is caused by direct repression of Fgf4 transcription by GATA factors (new Fig. 2B-D, new Fig. S4, lines 170 - 192). See our response to Reviewer #1, major point 2, for more details.

8. In Fig 2F statistical analysis between the re-seeded conditions is required for the conclusion that "the proportion of PrE-like cells systematically increased with cell density".
Replating itself appears to quite drastically impact lineage distribution. Do the authors have an explanation for this?

The p-value associated with this analysis referred to a test for a linear trend amongst the three re-seeded conditions following ANOVA in GraphPad Prism. We apologize that this has not been made clear in the original manuscript and have now added this information (lines 223, 224).

Replating disrupts the colony structure of the culture and therefore reduces the number of direct cell- cell contacts. The observation that replating drastically reduces the proportion of PrE-like cells is therefore perfectly in line with the overall conclusion from this section, namely that FGF signaling, which promotes PrE-like differentiation, is enhanced by cell-cell contacts. We now explicitly mention this explanation, alongside with a statistical test for the proportion of PrE-like cells between unperturbed and all re-seeded conditions, in lines 220 - 222 of the revised manuscript.

9. Fig 2G shows a key experiment illustrating the local effect of Fgf4 expression on first and second neighbours. The authors have investigated this effect using a Fgf-signalling reporter. Why did they not assay Gata6 expression in this assay instead of a Spry reporter? This would be the experiment to show that also Gata6 expressing cells (after transient Gata4 induction) are clustered around Fgf4 producing cells and be a strong piece of evidence to show that local Fgf4 signalling and cell-cell communication is indeed involved in cell identity proportioning. The cell lines required for this experiment (including Fgf4 mutant Gata4 inducible ESCs) appear to be available.

We have performed the suggested experiment by seeding a low number of labelled wild type cells to a culture of Fgf4 mutant inducible cells immediately after the end of a doxycycline pulse. As suspected by the Reviewer, after 16 h of differentiation GATA6-positive cells are largely confined to colonies that contain wild type cells, and often cluster around the wild type cells. This additional piece of evidence for local FGF4 signaling is shown in Fig. 3E, F and described in lines 240 - 244 of the revised manuscript.

10. The authors conclude from data in Fig 3A that proper cell type proportioning depends on initial Gata4 levels in Fgf4 mutants, in contrast to WT cells where the initial levels appear more irrelevant. Is 10ng/ml too high a dose? Would using a lower concentration (such as ~2ng/ml suggested by Fig 2D to give WT- like distribution) result in a complete rescue of cell lineage proportioning in this assay? Formally a control of adding additional Fgf4 to WT cells will also ne needed to control for a potential effect of exogenous Fgf4 addition.

© 2021. Published by The Company of Biologists under the terms of the Creative Commons Attribution License (https://creativecommons.org/licenses/by/4.0/).

To address this concern, we now show an experiment where we explore combinations of a range of induction times and FGF4 doses in the Fgf4 mutant (new Fig. S7, lines 255- 258 of the revised manuscript). This confirms the data from Fig. 2D of the original manuscript quoted by the Reviewer, that cell lineage proportions can be rescued to wild type levels by applying a lower dose of FGF4 following high GATA4-mCherry induction levels. However, this experiment also shows that cell type proportions in the Fgf4 mutant depend on induction levels at all FGF4 concentrations tested, and thus further support our conclusion that cell type proportioning in Fgf4 mutants depends on initial conditions.

The effects of adding FGF4 to wild type cells are shown in Fig. S2C and S3D and explicitly discussed in line 148 of the revised manuscript. The same control had also been performed for the experiments shown in Fig. 4A-C. We decided to omit it for clarity in the main figure, but now show it in the new Fig. S7C alongside the results of the corresponding simulations of the model (see our response to point 11).

11. Does the model in Fig 3E consider potentially varying doses of exogenous Fgf4? Can the model also predict what happens if Fgf4 is added to WT cells, as suggested above as control? In general, the value of this model is unclear. Figure 3E is near impossible to understand, no quantitative information is given.

To answer the Reviewer's questions, we have performed simulations of the mutant model with varying doses of FGF4 that we show in a new Fig. S7B of the revised manuscript. These simulations recapitulate the corresponding experimental results, namely that cell type proportions depend on initial conditions at all FGF concentrations tested (lines 284 - 287). We have also simulated addition of FGF4 to wild type cells. This shifts cell type proportions towards all cells becoming GATA-positive, again mirroring experimental results (new Fig. S7C,D, and lines 287 - 290).

The Reviewer's comment that the value of the model is unclear indicates to us that we have not explained in sufficient detail the conceptual differences between the behavior of the model in the wild type and the mutant case. As detailed in our response to Reviewer's comment 6. above, we have rewritten the text to more clearly contrast the two conditions, and hope that this clarifies the new insight that the model brings (lines 260 - 263 and 281 - 284).

To help the reader digest Fig. 4E, we have added separating lines similar to the gates of the flow cytometry data in panel A, and indicated the proportion of cells in the respective quadrants.

12. Fig4A: What were WT and Fgf4 mutant cells treated differently in this assay (8h vs 4h, respectively)?

The spatial arrangement of cell types in Fgf4 mutant cells has been assayed in two conditions that give similar cell type proportions as seen in the wild type, as motivated in lines 338 - 341. We decided to show the condition with 4 h induction followed by differentiation in the presence of 10 ng/ml FGF4 in the main Figure 5 because it is most similar to the condition that gives wild-type like cell type proportions in the Fgf4 mutant shown in the immediately preceding main Figure 4, while the condition that uses 8 h induction followed by differentiation in the presence of 2.5 ng/ml FGF4 refers back to the main Figure 2. We show both primary data and the complete analysis for the latter condition in Figures S9D and S11. Fig. S11 provides a direct comparison between the two conditions and clearly demonstrates that they show similar dynamics. We do not think that exchanging the two datasets between main and supplementary Figures will add value to the manuscript.

13. Does the interpretation that at 24h there is a difference in Fig 4C survive statistical scrutiny? Only few datapoints are shown and any apparent differences seem due to outliers rather than a shift in cluster radii. How often were these experiments independently repeated? This information is missing. In Fig 4B, I cannot appreciate any difference between cell lines.

We have performed statistical testing for the data shown in Fig. 5C. When comparing between wild type and mutant at each time point, we did not find any significant differences. We have therefore re-written the text accordingly (lines 342 - 344).

© 2021. Published by The Company of Biologists under the terms of the Creative Commons Attribution License (https://creativecommons.org/licenses/by/4.0/).

The information of independent experimental repeats has been added to the legend of Fig. 5A (lines 1023, 1024)

The similarities between wild type and Fgf4 mutant cells shown in Fig. 5B are not surprising and fully in line with the data in panel C, which shows that differences between time points are much more pronounced than differences between genotypes. However, we realize that the micrographs and analysis plots in Fig. 5A,B or the original manuscript were perhaps not fully representative of the aggregate behavior shown in panel C. In the revised manuscript, we therefore show data from more representative colonies in panels A and B.

Minor points:

a) More information on statistics should be given in the Figures and legends.

In the revised manuscript, we statistically compare the proportions of the Epi- and PrE-like cells for the different conditions in Figs 1, S2, S3, and 4 using Tukey's multiple comparisons test. These analyses support our main conclusions: In most cases, these cell type proportions do not significantly differ between GATA4-mCherry induction levels in wild type cells differentiated in N2B27 alone (Fig. 1 and Fig. 3), but they systematically change with induction levels in the presence of exogenous FGF4, both in wild type (Figs. S2 and S3), and Fgf4 mutant cells (Fig. 4). We have therefore decided to mention the results of these statistical tests in the main text rather than the Figures or legends.

b) Percentages should be indicated in the quadrants of the FACS plots of Fig 3A and E.

This is a good suggestion, we have added this information. See also our response to point 11 above.

c) What is the underlying evidence for the statement: "The specification of Epi- and PrE-like cells in ESCs shows both molecular and functional parallels to the patterning of the ICM of the mouse preimplantation embryo."

To address this point, we have changed the order or the argument in the respective section of the discussion. We start first compare cell differentiation in our ESC system with ICM patterning in the embryo (lines 405 ff), which leads us to conclude that "parallels between proportioning of Epi- and PrE- like cells in ESCs and the patterning of the ICM of the mouse preimplantation embryo suggests that similar mechanisms operate in both systems." (lines 417 - 419)

d) Fig 5C is difficult to interpret without a comprehensive decoding of colour information.

To facilitate interpretation of this panel, we have added a legend to decode the colour information of the traces (purple: VNP^{high}, cyan: VNP^{low})

Reviewer #2 (Significance (Required)):

This manuscript provides novel insights into the role of Fgf-mediated cell-cell communication to establish proper ratios of cell identities in a PrE-induction system. The authors provide some interesting data and interpretation. Overall, the significance is slightly impaired by the highly artificial nature of the studied cell fate specification event.

This manuscript will be of interest to readers working on early embryonic cell fate decision as well as researchers working on modelling of cellular processes.

My expertise lies in the field of cell fate decision and pluripotency.

Reviewer #3 (Evidence, reproducibility and clarity (Required)):

It is well established that FGF signalling plays a role in the partitioning of the Primitive Endoderm and Epiblast fates during preimplantation mammalian development. Recent work has shown that this fate decisions is associated with a mechanism that is able to maintain the proportions of the

© 2021. Published by The Company of Biologists under the terms of the Creative Commons Attribution License (https://creativecommons.org/licenses/by/4.0/).

two fates stable in the face of perturbations. Here, the authors address this mechanism and show that it is dependent on FGF signalling and associated with the fate decision. In the process they suggest and test a novel mechanism based on short range FGF signalling. A series of carefully designed and executed experiments, refine and provide evidence for the model. This is an original and important piece of work that will influence the field of pattern formation.

Overall the manuscript is well written but, at least from the perspective of this reviewer, there are places in which clarity can be improved.

Lines 104 and ff: the description of the dynamics of the different populations fater the GATA4 pulse, can be clarified. The reference to the double negative population emerging from the PrEnd population is not clear. It is stated that the proportion of these cells increased continuously and it said to be at the expense of the decrease of the PrEnd population whose variation is referred to as 'slightly declined". How can a slight decline fuel a steady increase in the double negative?

Also, what are these double negative? Could they be cells differentiating into embryonic lineages?

We realize from the comments of all three Reviewers on this paragraph that it was confusing and potentially misleading in the original manuscript. In our response to Reviewer #2, point 5., we describe in detail the changes that we have made in Fig. S1C, D and lines 109 - 114 of the revised manuscript to address this issue. Briefly, by applying an alternative gating strategy to the flow cytometry data shown in Fig. S1C, we show that the two cell types are well separated in the NANOG;GATA6 expression space from 40 h onwards, suggesting that cell fate assignment is complete and that changes of cell identity are rare beyond this point. Based on these observations, our interpretation of the decline in the GATA6- positive population is a lower proliferation rate compared to the GATA6-negative population.
The new gating strategy does not define a double-negative population. Instead, it reveals a progressive downregulation of NANOG expression in the GATA6-negative population. As pointed out by the Reviewer, this behavior is consistent with the differentiation along embryonic epiblast lineages, and therefore provides further support to our conclusion that "transient expression of GATA4-mCherry followed by 40 h of differentiation in defined, growth-factor-free medium subdivides an initially homogeneous culture into two cell types with Epiblast- and PrE-like characteristics." We have re-written lines 114 - 116 and 117 - 120 of the revised manuscript accordingly.

In Figure 1 and its discussion, it would be good to see a representation of the stability of the final proportions relative to the different initial conditions, a variation on 1E.

This is a good suggestion. In the revised manuscript, we have added a new panel to Fig. 1, in which we plot the final proportions of Epi- and PrE-like cells versus the GATA4-mCherry expression levels for the different induction times. This clearly illustrates that the final proportions of cell types are largely independent from the initial conditions (new Fig. 1F, and lines 133, 134).

Paragraph lines 182 and ff: the report that GATA4 expression is able to suppress FGF4 signalling, autonomously is, at least for this reviewer, a novel and important result and one that impinges on the understanding of the process. The authors should emphasize this.

Prompted by this comment and a similar comment from Reviewer #1, we have performed a series of experiments that provide further support for the direct regulation of Fgf4 expression by GATA factors; see our response to Reviewer #1, major point 2, for a detailed description. Briefly, we show that Fgf4 mRNA levels decrease rapidly upon induction of GATA4-mCherry, preceding NANOG protein downregulation (new Fig. 2B and Fig. S1A,B). Furthermore, we show that Fgf4 remains expressed in many cells upon culture for 40 h in N2B27 without doxycycline induction, even though NANOG is almost completely downregulated under these conditions (new Fig. 2C,D). A GATA6 binding site upstream of the Fgf4 gene that we identify in a published ChIP-seq dataset (new Fig. S4A,B) provides additional support for a direct regulation of Fgf4 through GATA factors, although functional testing of this site indicates that Fgf4 repression occurs through multiple, possibly redundant mechanisms (new Fig. 4C- E). The new data is discussed in lines 170 - 192 and 430 - 435 of the revised manuscript.

© 2021. Published by The Company of Biologists under the terms of the Creative Commons Attribution License (https://creativecommons.org/licenses/by/4.0/).

Paragraph lines 274 and ff (section on the involvement of FGF4 in the robustness of the process) needs some explanations. The derivation of the conclusion that 'recursive communication vis FGF4 underlies a population-level phenotype ...characterized by the differentiation of robust proportions of cell types..." from the experiments requires some unwrapping. It would be helpful if the authors could reason how the conclusion follows from the experiments.

To more clearly explain how this important conclusion follows from the results of the experiments shown in Fig. 4A - C, we have expanded on our reasoning in lines 260 - 263 of the revised manuscript. We explicitly state that the critical difference between mutant and the wild type cultures is “the connectivity of the cellular network: While cell differentiation in wild type cultures is coupled via FGF4, cells in Fgf4 mutant cultures take differentiation decisions largely autonomously”. We hope that this additional explanation makes it clear how the phenomenon of cell type proportioning is a consequence of cell-cell communication via FGF4. See also our response to Reviewer #2, points 6. and 11..

Their model does not seem to include the commonly agreed regulatory interaction between Nanog and FGF4, at least not directly, and it would be helpful if a reasoning could be provided for this decision.

In addition to suggesting a direct regulation of Fgf4 transcription by GATA factors, the new data in Fig. 2B-D also suggest that the commonly agreed positive influence of NANOG on Fgf4 transcription plays less of a role in ESCs. Specifically, Fgf4 mRNA is fully downregulated after 8 h of GATA4-mCherry induction, while NANOG is still present at this point. Conversely, Fgf4 remains expressed in cells transferred to N2B27 without a doxycycline pulse, even though NANOG is almost completely downregulated under these conditions. These data motivated us not to include a direct regulation between NANOG and Fgf4, as we discuss in lines 271/272, and 430 - 435 of the revised manuscript.

Reviewer #3 (Significance (Required)):

In this manuscript, Raina and colleagues use an Embryonic Stem (ES) cell based experimental system to address a central problem in developmental biology, namely the emergence of stable scaled populations of different cell fates. The experiments are elegant in design, carefully executed and the effort provides a solution to the problem: a novel mechanism based on short range FGF signalling that provides homeostatic control of relative cell populations. This is an important piece of work with sound conclusions that establishes a new paradigm in pattern formation whose implications are likely to lead to a reassessment of the role of FGF in different patterning paradigms. The experiments are quantitative and supported by a modelling effort based on a theoretical piece of work (Stanoev et al. 2021) which underpins the conclusion.

This manuscript will appeal to a wide audience including developmental and stem cell biologists as well as modellers.

My expertise cover the areas addressed in the manuscript.

Referees cross-commenting

It looks as if, with some nuances, we all agree on the value of the work. I do not have any issues with the comments of Reviewer 1, though I disagree that the model tested and improved here is similar to existing ones. While it is true that this work is related to a theory paper by some of the authors, the experimental test and resulting conclusions are very important. On the other hand, I am very surprised by the comments of Reviewer 2 who, after conceding the value and potential significance of the work, raises a list of queries, largely small details and opinions rather than points of substantial concerns, hinting at a need for the authors to perform extra work and analysis that will not change the conclusions of the manuscript. Some of this e.g. #9 would be a nice piece of additional evidence, but more an adornment than a necessary piece of additional evidence. The main problem of this reviewer is the lack of appreciation of what they define as 'highly artificial nature' of the study without providing any reason for why such experiments (very common in developmental biology) can lead to misleading conclusions. It seems

© 2021. Published by The Company of Biologists under the terms of the Creative Commons Attribution License (https://creativecommons.org/licenses/by/4.0/).

to me that most, if not all, of their significant concerns can be dealt with in a rebuttal or by altering the text, either to discuss the issues raised, to clarify the points or qualify the conclusions.

Second decision letter

MS ID#: DEVELOP/2021/199926

MS TITLE: Cell-cell communication through FGF4 generates and maintains robust proportions of differentiated cell types in embryonic stem cells

AUTHORS: Dhruv Raina, Azra Bahadori, Angel Stanoev, Michelle Protzek, Aneta Koseska, and Christian Schroter
ARTICLE TYPE: Research Article

I am happy to tell you that your manuscript has been accepted for publication in Development, pending our standard ethics checks.

Reviewer 1

Advance summary and potential significance to field

This work, using a well-controlled ESC differentiation protocol, provides novel evidence that the spatial range of FGF signaling activity plays an important role in controlling the adoption of PE and Epiblast fates. Despite the intrinsic limits of the in-vitro systems adopted, the conclusion of this work delineate a model that will help understand the mechanisms regulating this crucial cell fate transition during early development.

Comments for the author

In this revised version of their manuscript, Raina et al. have satisfactorily addressed all major concerns raised in my previous comments. Specifically, the authors performed better-controlled experiments and discuss more clearly how the presence or absence of exogenously added FGF affects the dependence of the experimental system on the initial levels of Gata4 induction. Most importantly efforts have been made to present some evidence of a direct control of Fgf4 expression by GATA factors, including performing a clearer in-situ analysis of Fgf4 expression shortly after acute GATA4 induction, and attempting to identify potential regulatory element through which such control might be exerted. Although not providing irrefutable evidence, the rapid responses described support the existence of a direct transcriptional effect, and strengthen the main conclusion of the work: under the regulation of GATA factors, short-range cell-to-cell communication via FGF robustly balances the acquisition of PE and Epiblasts fates.

Reviewer 2

Advance summary and potential significance to field

see original submission

Comments for the author

In their revised manuscript Raina et al addressed most of my concerns, have added new data and provide a much-improved manuscript.
The authors now disclose the artificial nature of their experimental system in the discussion. However, the conclusion that ‘the parallels between proportioning of Epi- and PrE-like cells in ESCs and the patterning of the ICM of the mouse preimplantation embryo suggests that similar mechanisms operate in both systems.’, is still overly speculative. What exactly are the parallels

© 2021. Published by The Company of Biologists under the terms of the Creative Commons Attribution License (https://creativecommons.org/licenses/by/4.0/).

that make the authors arrive at this conclusion? I suggest a more careful wording in the discussion, or to clearly label speculation as such.
I still disagree with the use of the word 'differentiation' for Gata4 induced establishment of PrE-like cells from ESCs. This is a cell fate decision that would not occur during normal development and is therefore not part of the differentiation decisions available for WT ESCs. However, to the informed reader the fine distinction between developmentally relevant differentiation and induced acquisition of cell identity will be sufficiently clear; although clarity in language would avoid any potential misunderstandings for readers not in the field. Regardless, this disagreement on semantics should not delay publication.

© 2021. Published by The Company of Biologists under the terms of the Creative Commons Attribution License (https://creativecommons.org/licenses/by/4.0/).
